# Exploring the Protective Effects and Mechanism of Crocetin From Saffron Against NAFLD by Network Pharmacology and Experimental Validation

**DOI:** 10.3389/fmed.2021.681391

**Published:** 2021-06-09

**Authors:** Zijin Xu, Susu Lin, Junjie Gong, Peishi Feng, Yifeng Cao, Qiaoqiao Li, Yuli Jiang, Ya You, Yingpeng Tong, Ping Wang

**Affiliations:** ^1^College of Pharmaceutical Sciences, Zhejiang University of Technology, Hangzhou, China; ^2^School of Life Sciences, Taizhou University, Taizhou, China

**Keywords:** saffron, crocetin, NAFLD, network pharmacology, Nrf2, HO-1

## Abstract

**Background:** Non-alcoholic fatty liver disease (NAFLD) is a burgeoning health problem but no drug has been approved for its treatment. Animal experiments and clinical trials have demonstrated the beneficial of saffron on NAFLD. However, the bioactive ingredients and therapeutic targets of saffron on NAFLD are unclear.

**Purpose:** This study aimed to identify the bioactive ingredients of saffron responsible for its effects on NAFLD and explore its therapy targets through network pharmacology combined with experimental tests.

**Methods:** Various network databases were searched to identify bioactive ingredients of saffron and identify NAFLD-related targets. Gene ontology (GO) and Kyoto Encyclopedia of Genes and Genomes (KEGG) enrichment were conducted to enrich functions and molecular pathways of common targets and the STRING database was used to establish a protein-protein interaction network (PPI). The effect of crocetin (CCT) on NAFLD was evaluated in a mouse model of NAFLD by measuring the biomarkers of lipid, liver and renal function, oxidative stress, and inflammation. Liver histopathology was performed to evaluate liver injury. Nuclear factor erythroid-related factor (Nrf2) and hemeoxygenase-1 (HO-1) were examined to elucidate underlying mechanism for the protective effect of saffron against NAFLD.

**Results:** A total of nine bioactive ingredients of saffron, including CCT, with 206 common targets showed therapeutic effects on NAFLD. Oxidative stress and diabetes related signaling pathways were identified as the critical signaling pathways mediating the therapeutic effects of the active bioactive ingredients on NAFLD. Treatment with CCT significantly reduced the activities of aspartate aminotransferase (AST), alanine transaminase (ALT), and the levels of total cholesterol (TC), triglyceride (TG), malondialdehyde (MDA), blood urea nitrogen (BUN), creatinine (CR), and uric acid (UA). CCT significantly increased the activities of superoxide dismutase (SOD), and catalase (CAT). Histological analysis showed that CCT suppressed high-fat diet (HFD) induced fat accumulation, steatohepatitis, and renal dysfunctions. Results of ELISA assay showed that CCT decreased the expression of tumor necrosis factor-α (TNF-α), interleukin-6 (IL-6), interleukin-1β (IL-1β), and increased the expression of HO-1 and Nrf2.

**Conclusion:** This study shows that CCT is a potential bioactive ingredient of saffron that treats NAFLD. Its mechanism of action involves suppressing of oxidative stress, mitigating inflammation, and upregulating Nrf2 and HO-1 expression.

## Introduction

Non-alcoholic fatty liver disease (NAFLD) is a clinical syndrome characterized by lipid accumulation in the hepatocyte, steatosis, cellular injury, and hepatic infiltration of inflammatory cells (1, 2). The global prevalence of NAFLD is estimated to be ~25% (Younossi et al., 2018). In recent years, NAFLD pathogenesis has been explained using the “one-hit,” “two-hit,” and “multiple-hit” hypotheses (3). Traditionally, a high-fat diet (HFD) was considered an important factor in NAFLD development (4). However, growing evidence has revealed that other factors such as race, ethnicity, gender, age, and genetic pre-disposition are associated with NAFLD (5–7). Further elucidation of the molecular mechanism of NAFLD has revealed that oxidative stress, endoplasmic reticulum stress, perturbation of autophagy, mitochondrial dysfunction, hepatocellular apoptosis, and inflammatory responses cause liver damage in NAFLD (8). Among them, the role of oxidative stress in the pathogenesis of NAFLD has attracted significant attention (9). Previous studies have suggested multiple molecular pathways related to oxidative stress that is involved in NAFLD, including IRs-1/Akt, CYP2E1/JNK, SIRT1/SIRT3-FOXO3a, A(10)MPK/Nrf2, Nrf2/HO-1, and AMPK signaling pathways (10–13).

Herbal medicine has been used in NAFLD treatment, specifically on multiple mechanisms underlying lipid metabolism and inflammation (14). Saffron is one of the most expensive herbs in the world, obtained from dried stigmas of *Crocus sativus* L. (15). Islamic traditional medicine literature introduced saffron as an astringent, resolvent, and concoctive drug, with therapeutic effects on gastro-hepatoprotective, urogenital disorders, antidepressants, ocular disorders, etc. (16). The biological activity of saffron has been widely studied, specifically focusing on its immunomodulatory, anti-inflammatory, and antioxidant activities, and in the treatment of cardiovascular and depression-anxiety (17–20). Besides, recent research demonstrates a hepatoprotective effect of saffron in diabetic rats (21). Results from clinical trials have indicated that saffron supplementation has beneficial effects on the serum levels of inflammatory, oxidative stress, and adipokines biomarkers in patients with NAFLD (22). Existing research provides evidence on the effect of saffron in the treatment of NAFLD, however, further studies are needed to better elucidate the main bioactive ingredients and signaling pathways.

**Figure 1:**
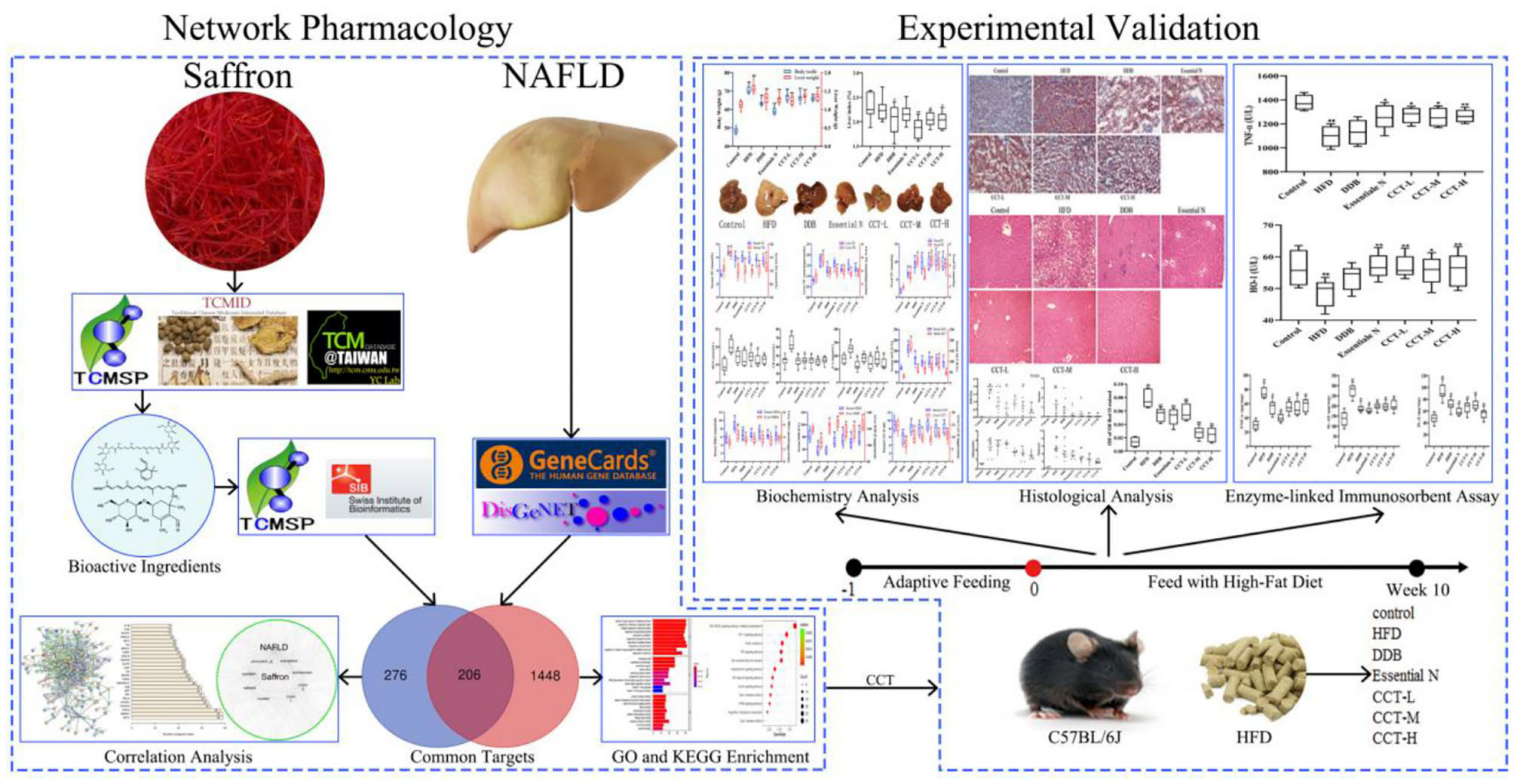
The schematic diagram of the present study.

In the current study, network pharmacology was used to predict the main bioactive ingredients, potential therapeutic targets, and key pathways responsible for the effect of saffron in the treatment of NAFLD. Subsequently, the effects and possible signaling pathways of CCT on NAFLD treatment were evaluated in HFD fed mice. The schematic diagram of this study is shown in Figure 1.

## Materials and Methods

### Herb Materials, Reagents, and Animals

Saffron samples were collected from Shenghuo Agricultural Development Co., Ltd (Hangzhou, China). CCT reference substance was purchased from Chengdu Desite Biotechnology Co., Ltd (the content of CCT ≥ 98%, Chengdu, China). The total cholesterol (TC) assay kit, Triglyceride (TG) assay kit, Urea (BUN) assay kit, Creatinine (Cr) assay kit, Uric acid (UA) assay kit, Malondialdehyde (MDA) assay kit, Catalase (CAT) assay kit, Superoxide Dismutase (SOD) assay kit, Oil Red O dye, Haematoxylin, and Eosin dye were purchased from Jiancheng Biotechnology Institute Company (Nanjing, China). RIPA lysis buffer, Bicinchonininc acid (BCA) protein determination kit, and Nuclear and Cytoplasmic Protein Extraction kit were purchased from Beyotime Company (Shanghai, China). Nuclear factor erythroid-related factor (Nrf2) transcription factor assay kit, Hemeoxygenase-1 (HO-1) ELISA kit, Tumor necrosis factor-α (TNF-α) ELISA kit, Interleukin-1β (IL-1β) ELISA kit and Interleukin-6 (IL-6) ELISA kit were purchased from Mlbio Biotechnology Company Limited (Shanghai, China). The HFD (saccharose 20%, lard 15%, cholesterol 1.2%, sodium cholate 0.2%) was purchased from Trophic Animal Feed High-Tech Co., Ltd. (Nantong, China). C57BL/6J mice (male, 5 months old, bodyweight 27 ± 2 g; Shanghai Slac Laboratory Animal CO., Ltd., SCXK 2012-0002) were housed individually in ventilated cages (five per cage) and controlled environmental conditions in a clean grade facility at Zhejiang University of Technology (temperature 22–25°C; relative humidity 60%). The mice had free access to standard laboratory chow and tap water and were maintained under normal conditions of a 12/12 h light/dark cycle. All experiments were conducted at the Animal experimental research Center of Zhejiang University of Technology. The study was approved by the Animal Protection Research Ethics Committee of Zhejiang University of Technology (20190603064).

### Screening for Bioactive Ingredients in Saffron

The bioactive ingredients of Saffron were obtained from the following databases: the Traditional Chinese Medicine Systems Pharmacology (TCMSP, http://tcmspw.com/tcmsp.php) (23), TCM database @taiwan (http://tcm.cmu.edu.tw) (24), and Traditional Chinese Medicine Integrated Database (TCMID, (http://www.megabionet.org/tcmid/) (25). The parameters oral bioavailability (OB) ≥ 30% and drug-likeness (DL) ≥ 0.18 were set as thresholds to identify the potential bioactive ingredients in TCMSP (26). To obtain comprehensive information of bioactive ingredients, recent literature and relevant international standards such as ISO 3632-1:2011 were reviewed (27–30).

### Correlation Analysis Between Saffron and NAFLD-Related Targets

The corresponding targets of the bioactive ingredients of saffron were collected from the TCMSP database and SwissTargetPrediction database (http://www.swisstargetprediction.ch/). The GeneCards database (http://www.genecards.org/) and DisGeNET database (https://www.disgenet.org/) were searched using the terms “NAFLD” or “non-alcoholic fatty liver disease” (UMLS CUI: C0400966) to identify the potential therapeutic targets of NAFLD. The UniProt database (https://www.uniprot.org/) was used to standardize gene information, and further to remove duplicate genes and pseudogenes. The common targets of the bioactive ingredients were manually screened and the interaction network was visualized using Cytoscape 3.7.2. software. Gene Ontology (GO) and Kyoto Encyclopedia of Genes and Genomes (KEGG) pathway enrichment analyses were performed by using the R package “clusterProfile.” The visualization and integration of enrichment results were carried out by using Enrichlot and ggplot2 R package. The above steps were completed by R software 3.6.2 (x64) (31). Data were presented using bar charts and a bubble chart. *P-*value < 0.05 or *Q-*value < 0.05 were considered statistically significant. The construction of the protein-protein interaction (PPI) network for the common targets was generated through the String database (https://string-db.org/cgi/input.pl). Subsequently, the parameters of the PPI network, including the node degree (Degree), closeness centrality (CC), and betweenness centrality (BC) were calculated using the Network Analyzer tool function of the String database, and the results presented in a bar chart and 3D scatter plot.

### Extraction and Purification of CCT

**Figure 2:**
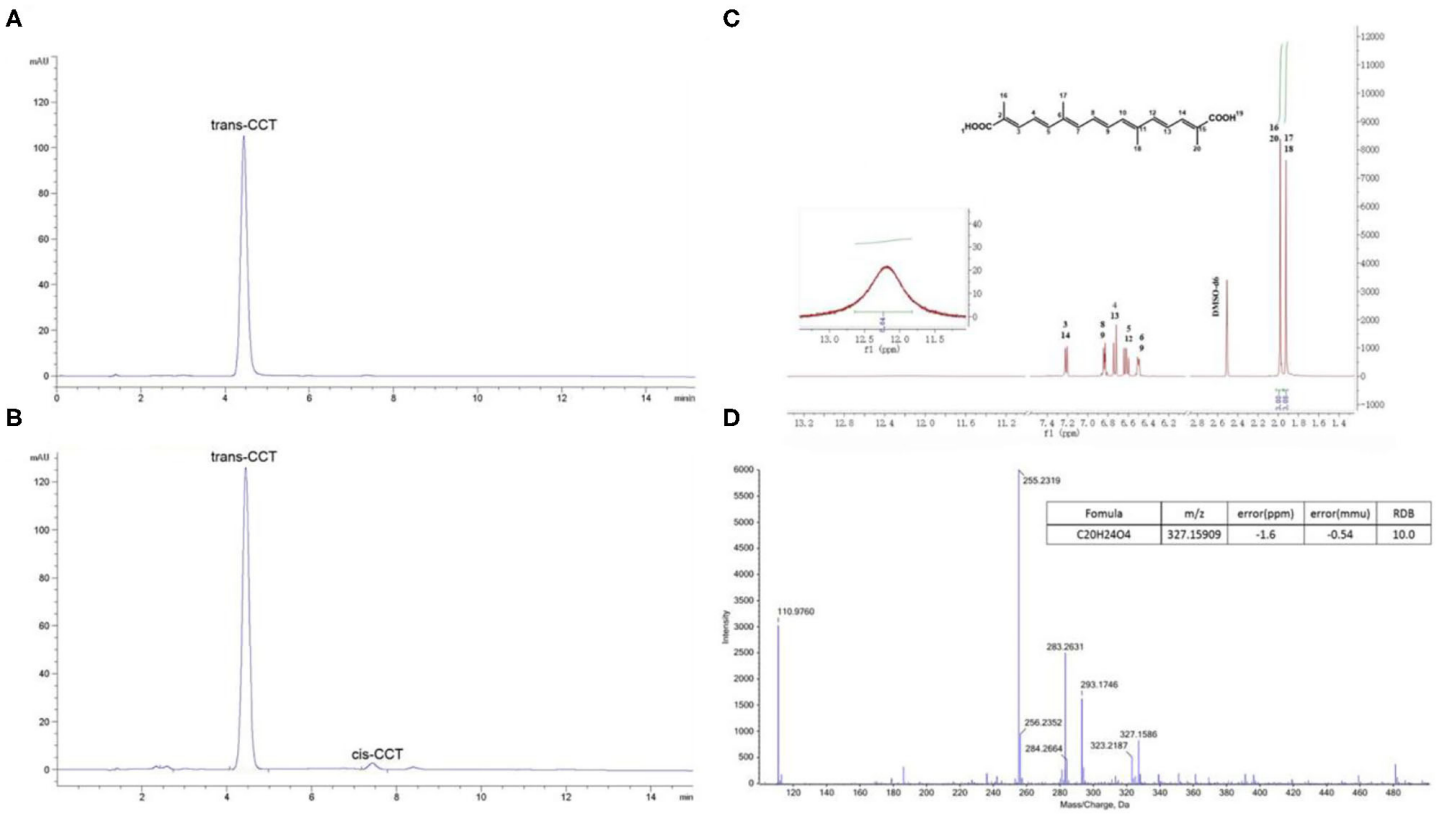
The purity and structure analysis of CCT. **(A)** HPLC chromatogram of CCT reference substance. **(B)** HPLC chromatogram of CCT. **(C)** ^1^H-NMR of CCT. **(D)** (-) ESI-MS/MS of CCT.

The extraction and purification of CCT from saffron were performed as reported in our previous research, but with slight modifications (32). Ultrasonic technology was applied in the extraction of CCT and 65% ethanol was used as the extracting solvent. The optimal extraction parameters were as follows: extraction temperature was 50°C, a 1:14 raw material to liquid ratio and ultrasonic power of 100 W. Extraction was performed 3 times for 1 h each. The D101 macroporous resin was used in extract purification and the purification parameters were as follows: eluents were water, 25% ethanol and 60% ethanol, elution volume of 8-bed volumes. The water and 25% ethanol eluent were discarded, while the 60% ethanol eluent was collected. NaOH solution (2 M) was used to adjust the pH of the solution to about 12, and the mixture was allowed to stand at room temperature for 12 h. H_2_SO_4_ solution (1 M) was used to adjust the pH to about 2, and the mixture precipitated overnight at 4°C. The sediment, which was a crude extract of CCT was collected by centrifugation. The crude extract was washed twice using methanol and 0.5% H_2_SO_4_ and washed twice with double-distilled water. Finally, the purified CCT was obtained by centrifugation and dried overnight in a vacuum oven at 60°C. The purity and structure of CCT were elucidated using HPLC (Figures 2A,B), ^1^H-NMR (Figure 2C) (600 MHz) and (-) ESI-MS/MS (Figure 2D) as shown in Figure 2.

### Animal Treatment and Sample Collection

After 7 days of adaptive feeding, mice were randomly assigned to seven groups (*n* = 10 in each group) as follows: (1) Mice in the control group (control) were fed normal chow diets and given 0.5% sodium carboxyl methyl cellulose (CMC-Na) daily by oral gavage; (2) The high-fat diet group (HFD) mice were fed with HFD and given 0.5% CMC-Na daily by oral gavage; (3) The biphenyldimethylesterate group (DDB) mice were fed with HFD and given 200 mg/kg of biphenyldimethylesterate daily by oral gavage; (4) The Essentiale N group (Essential N) mice were fed with HFD and given 200 mg/kg of Essentiale N daily by oral gavage; (5) The Low-dose CCT group (CCT-L) mice were fed with HFD and given 10 mg/kg of CCT daily by oral gavage; (6) The Medium-dose CCT group (CCT-M) mice were fed with HFD and given 30 mg/kg of CCT daily by oral gavage; (7) The High-dose CCT group (CCT-H) mice were fed with HFD and given 50 mg/kg of CCT daily by oral gavage. DDB and Essential N, two drugs previously shown to be effective in the treatment of NAFLD and liver injury, were used as positive controls during the validation experiment (33–35).

The body weight was recorded and blood samples were collected from the mice orbit after an overnight fast at 2, 4, 6, and 10 weeks. All blood samples were centrifuged (Thermo, American) at 3,500 rpm for 15 min and the supernatant was collected and stored at −80°C for further analysis. Mice were isolated into single cages for 4 h for collection of feces 10 weeks after the last gavage dose. The mice were sacrificed to collect the liver tissues. The liver weight was measured and the liver index calculated using the following formula: liver index = liver weight/body weight ×100% (g/g). Each of the liver samples was divided in two; one portion was frozen and stored at −80°C for further analysis and the other portion was fixed in 10% neutralized formalin for subsequent paraffin embedding and histological analysis.

### Biochemistry Analysis

Fasting serum AST, ALT, TC, TG, BUN, CR, and UA were analyzed using an automatic biochemical analyzer (model 7600; Hitachi Ltd., Japan). Feces and liver samples were placed in normal saline and homogenized using a tissue homogenizer (Scientz-48, Ningbo, China) for 60 s at 60 Hz. The supernatants were obtained by centrifugation for TC and TG analysis. MDA, CAT, and SOD in serum and homogenized liver tissue were analyzed using commercial assay kits according to the manufacturer's instructions.

### Histological Analysis

Haematoxylin and eosin staining (H&E) and Oil Red O staining were used to evaluate liver histopathology. Briefly, liver specimens fixed in 10% neutral formalin were embedded in paraffin, sliced at 5 μm thickness, and stained with haematoxylin and eosin. The NAFLD activity score (NAS) ranged from 0 to 8 was used to evaluate histological liver damage. More specifically, NAS includes three histological scores as follows: steatosis (0–3), lobular inflammation (0–3), and ballooning degeneration (0–2) (36). Hepatic lipid accumulation was determined using Oil Red O staining. The images were captured using an inverted optical microscope (Olympus IX81, Japan, magnification, ×200). Isopropyl alcohol was used to elute Oil red O stain and quantification was performed at 535 nm to determine the degree of lipid deposition. Data were expressed as the fold change to control.

### Inflammation Biochemical Assays

The levels of TNF-α, IL-1β, and IL-6 in the liver of each group were analyzed using commercial enzyme-linked immunosorbent assay (ELISA) kits according to the manufactures' instructions. The standard curve was used to calculate the concentration.

Enzyme-linked Immunosorbent Assay of Nrf2 and HO-1Nrf2 and HO-1 were determined by an enzyme-linked immunosorbent assay as described below. Part of the liver was first taken for total protein extraction by RIPA lysis buffer and the total protein concentration was determined by BCA protein determination kit. Nuclear extracts were isolated with a Nuclear and Cytoplasmic Protein Extraction kit and further used for the determination of Nrf2 and HO-1 by using commercial kits according to the manufactures' instructions. The standard curve was used to calculate the concentration.

### Statistical Analysis

All data in this study were presented as means ± SEM and analyzed using SPSS 17.0 statistical software. One-way ANOVA was used to statistically analyze the data. *P* < 0.05 was considered to be statistically significant (37).

## Results

### Bioactive Ingredients of Saffron

A total of 70 bioactive ingredients were retrieved from TCMSP, TCM @taiwan, and TCMID databases. However, according to the screening criteria of OB (≥30%) and DL (≥0.18), 5 bioactive ingredients were identified, including quercetin, kaempferol, isorhamnetin, CCT, and n-heptanal. Besides, another four bioactive ingredients were identified from the literature and included in the final results for subsequent analysis and included, crocin I, crocin II, safranal, and picrocrocin (27–30). Detailed information on the bioactive ingredients is listed in Table 1. n-heptanal was not included in the follow-up analysis because of its unclear structure and chemical abstracts service (CAS) number.

**Table 1:** The detailed information and parameters of nine bioactive ingredients of saffron.

**ID**	**Compound**	**OB(%)**	**DL**	**Molecular weight**
MOL001389	n-heptanal	79.74	0.59	352.42
MOL001406	Crocetin	35.30	0.26	328.44
MOL000354	Isorhamnetin	49.60	0.31	316.28
MOL000422	Kaempferol	41.88	0.24	286.25
MOL000098	Quercetin	46.43	0.28	302.25
MOL001409	Picrocrocin	33.71	0.04	168.26
MOL001405	Crocin I	2.54	0.12	977.08
MOL001407	Crocin II	1.65	0.21	814.92
MOL000720	Safranal	39.56	0.04	150.24

### NAFLD-Related and Bioactive Ingredients of Saffron Targets

**Figure 3:**
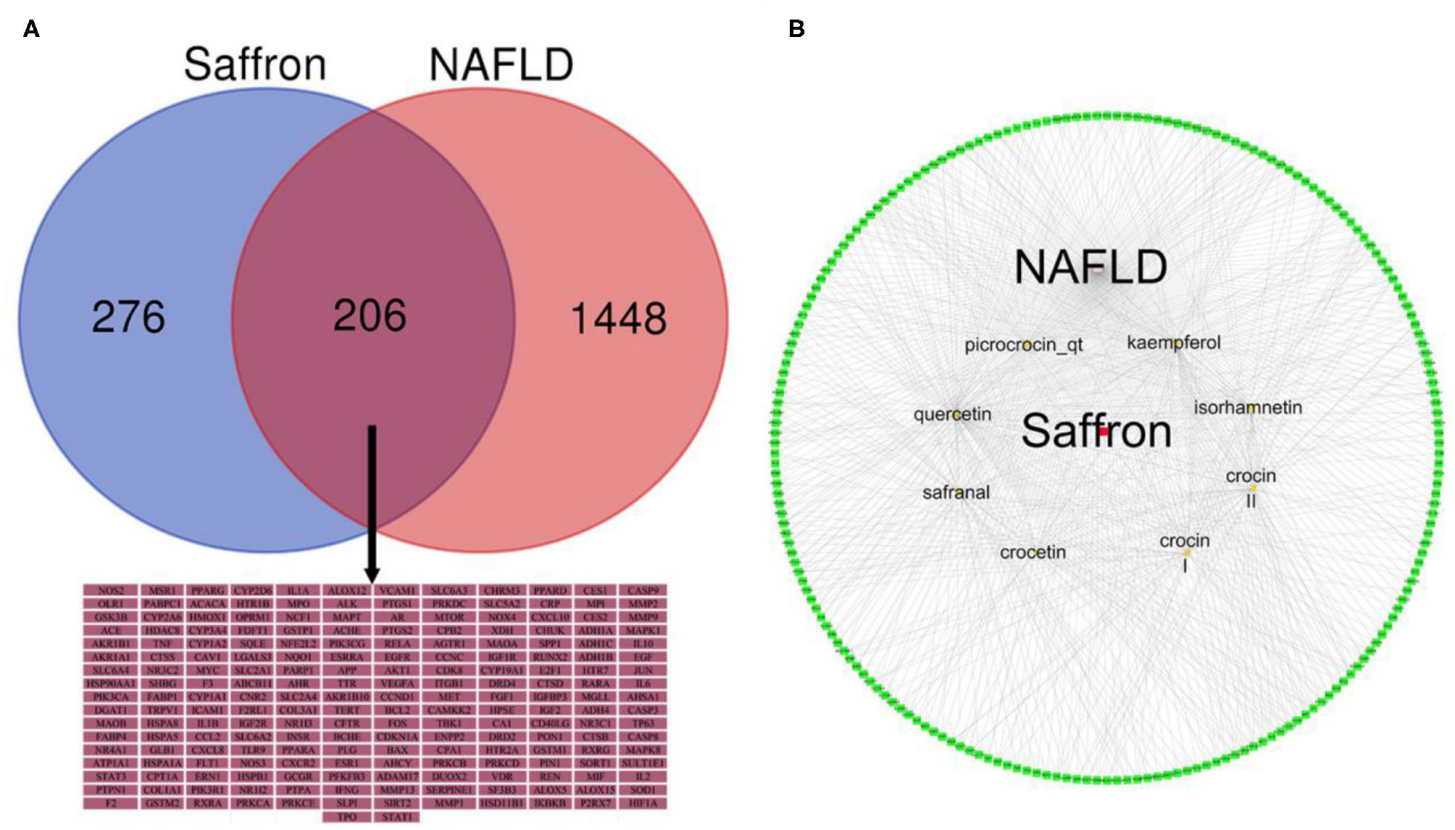
**(A)** Venn diagram of the targets of Saffron in NAFLD. **(B)** A network of bioactive ingredients and NAFLD-related targets. The red node represent saffron and the eight yellow nodes represent the bioactive ingredients in saffron; the green nodes represent common targets of the bioactive ingredients of saffron and NAFLD-related. The edges denote that nodes can interact with each other.

A total of 167 corresponding targets of the bioactive ingredients of saffron were extracted from TCMSP. However, two similar bioactive ingredients were identified as having similar targets in the SwissTargetPrediction database, so the number of targets extracted in the SwissTargetPrediction database was up to 800. A total of 485 targets of the bioactive ingredients of saffron were identified after merging and removing the duplicates using two databases. A total of 1,058 NAFLD-related targets with a cut-off value larger than 20 were obtained from the DisGeNet database, while 1,017 targets were obtained from the Genecards database. Finally, a total of 1,654 NAFLD-related targets were identified after merging and removing the duplicate records from the two databases. After the manual screening, the common targets of the bioactive ingredients of saffron and NAFLD-related were limited to 206 as shown in Figure 3A. More detailed information of 206 common targets was listed in Supplementary Table 1. Thereafter, the bioactive ingredients targets-NAFLD-related targets network was constructed as shown in Figure 3B. The bioactive ingredients of saffron were found to interfere with multiple therapeutic targets related to NAFLD. The number of NAFLD-related targets associated with quercetin, kaempferol, safranal, crocin I, crocin II, and CCT was 106, 63, 49, 41, 39, and 38, respectively, while the total number of NAFLD-related targets associated with crocin I, crocin II and CCT was 118. These findings indicated that CCT and its glucosyl ester derivatives (crocin I and crocin II) play an important role in the treatment of NAFLD.

### GO and KEGG Enrichment Analysis

**Figure 4:**
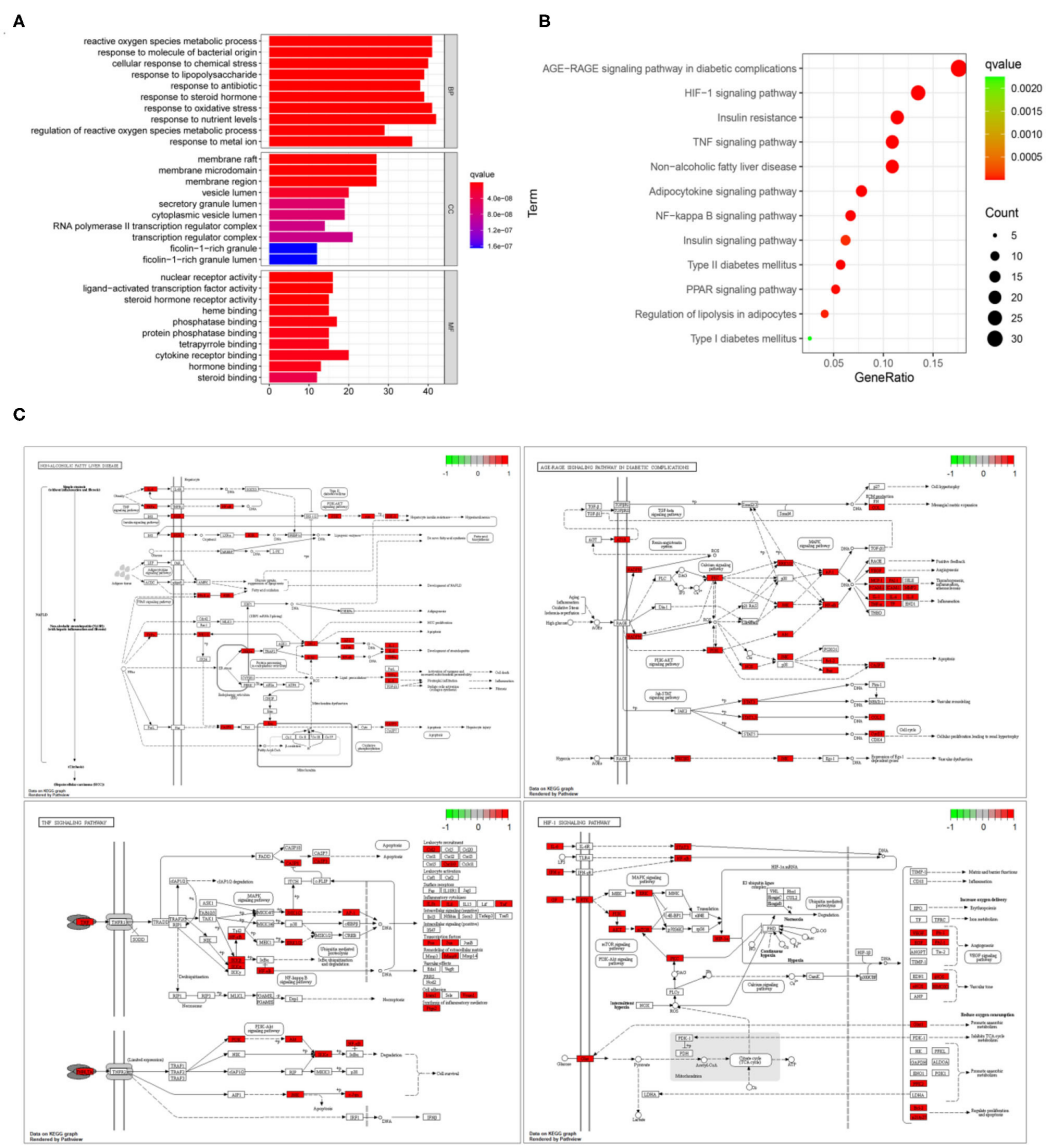
GO and KEGG enrichment analysis. **(A)** GO enrichment analysis of the 206 common targets associated with NAFLD. The *X* -axis represents significant enrichment in the counts of these terms; The *Y*-axis showing the categories of biological process (BP), cellular component (CC), and molecular function (MF) in the GO of the target genes (*P* < 0.05). **(B)** KEGG enrichment analysis. The *X*-axis showing the counts of the target symbols in each pathway; the *Y*-axis showing the main pathways (*P* < 0.05). **(C)** The specific position and function of common targets in in signaling pathways. The red region represents the specific position of these common targets in the pathways.

The GO enrichment analysis of 206 common targets was shown in Figure 4A. The key biological processes implicated in the treatment of NAFLD, included reactive oxygen species, metabolic process, response to molecules of bacterial origin, cellular response to chemical stress, response to lipopolysaccharide, response to an antibiotic, response to a steroid hormone, etc. In terms of cellular components, they were involved in membrane raft, membrane microdomain, membrane region, vesicle lumen, secretory granule lumen, and cytoplasmic vesicle lumen, etc. Additionally, nuclear receptor activity, ligand-activated transcription factor activity, steroid hormone receptor activity, heme binding, phosphatase binding, and protein phosphatase binding were associated with the molecular functions in the treatment of NAFLD. Figure 4B was a simplified bubble chart showing KEGG enrichment, which showed that the underlying mechanism of NAFLD mainly involved diabetes-related pathways, apoptosis, oxidative stress, and inflammatory pathways. For clearer presentation, the specific position and function of common targets are colored red in the signaling pathway in Figure 4C and the specific targets in signaling pathways were listed in Supplementary Table 2.

### PPT Network of Common Targets

**Figure 5:**
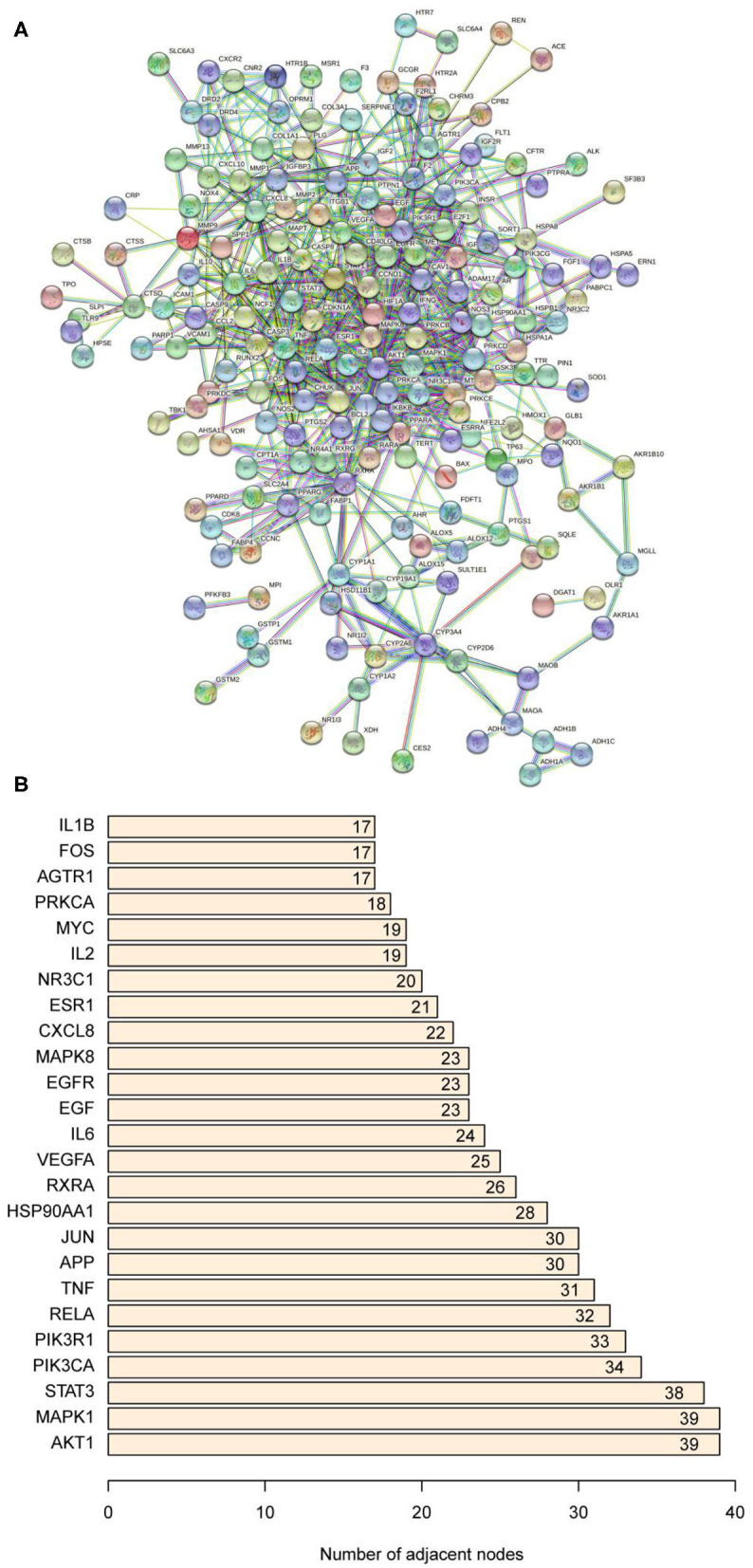
**(A)** Protein-protein interaction network. **(B)** The bar plot of the protein-protein interaction network. The *X*-axis represents the number of neighboring proteins of the target protein. The *Y*-axis represents the target protein.

The PPI network was constructed based on the NAFLD-related and bioactive ingredients of saffron targets. As shown in Figure 5A, the PPI network contained 184 nodes and 1,594 edges. The light blue edges and lavender edges represent the known interactions from curated databases and experimentally determined, respectively. The green edges, red edges, and dark blue edges represent predicted interactions with the gene neighborhood, gene fusions, and gene co-occurrence, respectively. The yellow edges, black edges, and light blue edges represent predicted interactions from textmining, co-expresson, and protein homology, respectively. Figure 5B showed that the top 10 core targets were AKT1, MAPK1, STAT3, PIK3CA, PIK3R1, RELA, TNF, APP, JUN, and HSP90AA1. Details of the PPI network are shown in Supplementary Table 3.

### Effects of CCT on Body Weight, Liver Weight, and Liver Index

**Figure 6:**
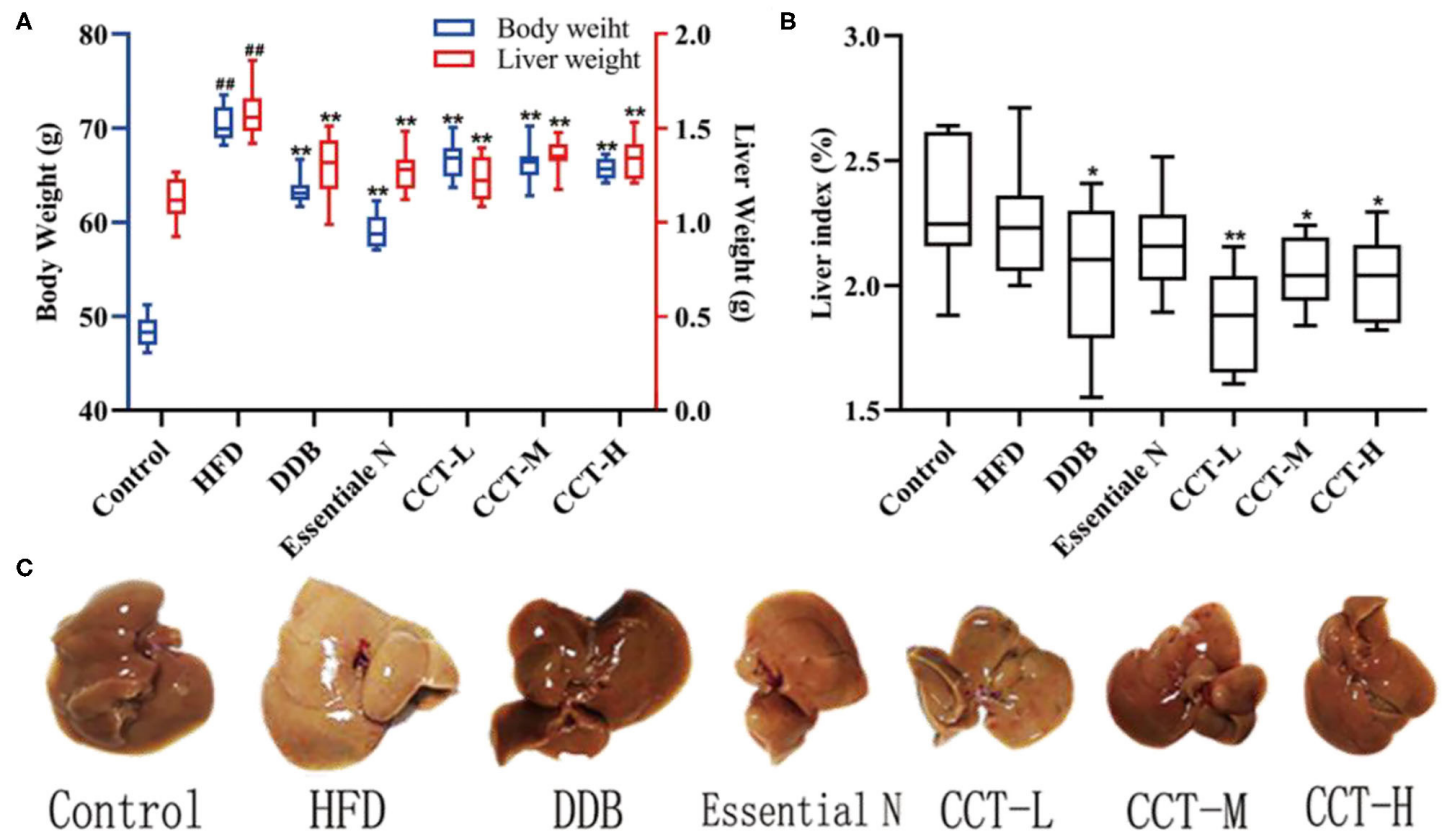
**(A)** Body weight and liver weight. **(B)** Liver index. **(C)** Liver photographs. ^##^*P* < 0.01 vs. Control group; *P < 0.05, **P < 0.01 vs. HFD group.

Compared with the control group, the body weight gain of the HDF group significantly increased at week 10. CCT was found to cause a reduction in body weight gain induced by HFD. In HFD mice, the liver weight was significantly increased. Except for the Essentiale N group, the liver index in all treatment groups significantly decreased compared with the HDF group (Figures 6A,B). The liver of the HDF group was canary yellow and its surface had a distinct white dot shape representing hepatic steatosis. In contrast, the liver of the DDB group, CCT-M group, and CCT-H group was significantly improved as shown in Figure 6C.

### Effects of CCT on Lipid, Liver, and Renal Function Biomarkers

**Figure 7:**
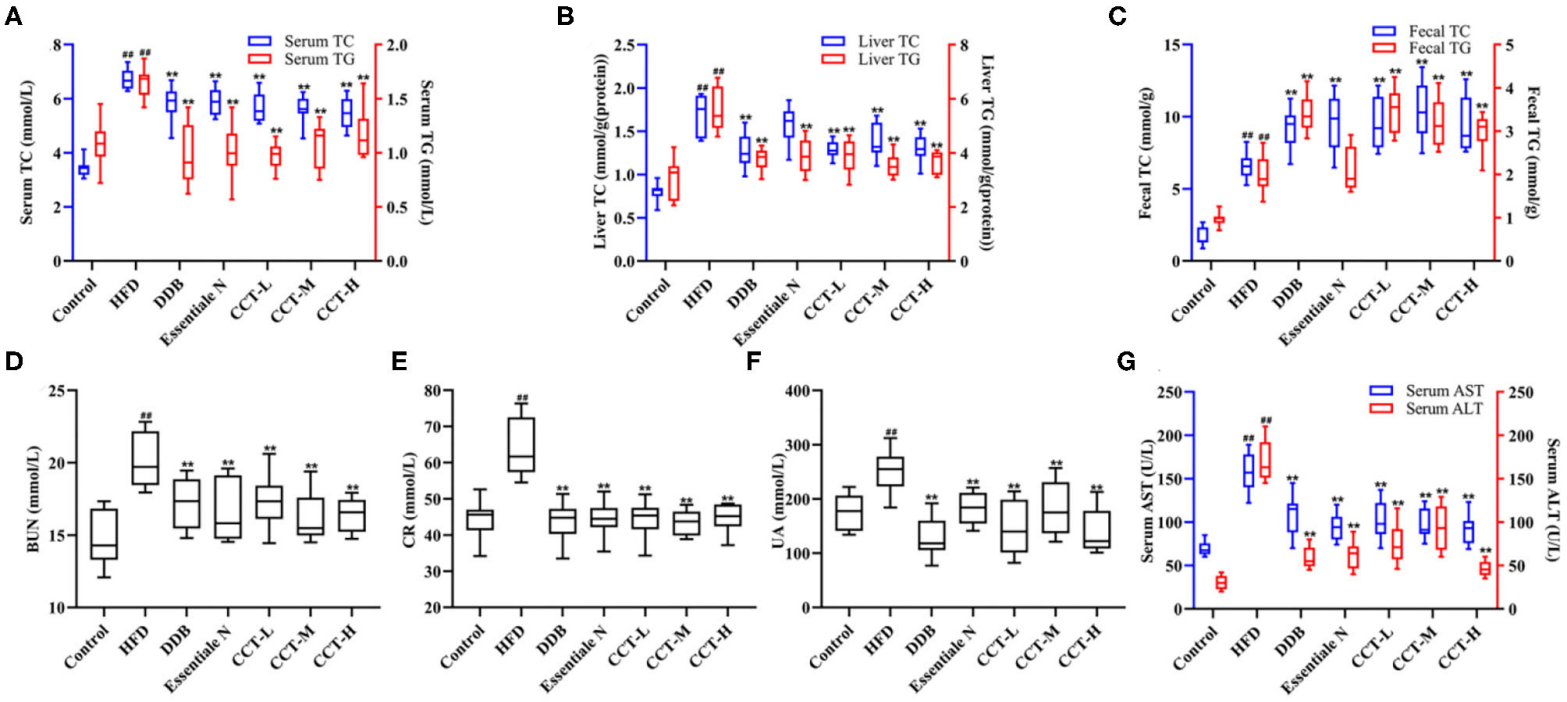
Effects of CCT on lipid, liver and renal function biomarkers. **(A)** Effects of CCT on serum TC and TG. **(B)** Effects of CCT on liver TC and TG. **(C)** Effects of CCT on fecal TC and TG. **(D)** Effects of CCT on BUN. **(E)** Effects of CCT on CR. **(F)** Effects of CCT on UA. **(G)** Effects of CCT on serum AST and ALT. ^##^*P* < 0.01 vs. Control group; **P < 0.01 vs. HFD group.

As shown in Figure 7, the levels of serum and liver TC and TG were significantly increased in the HFD group, while were significantly decreased in all treatment groups compared with the HDF group (Figures 7A,B). The levels of fecal TC and TG were significantly increased in three dose groups of CCT compared with the HDF group (Figure 7C). These findings indicated that CCT can increase lipid excretion to alleviate lipid accumulation caused by NAFLD. The activities of ALT and AST, two markers of liver function, were also significantly increased in the HDF group compared with the control group. The effect of CCT in reducing ALT and AST activities was observed after treatment, and also in the DBB and Essential N groups (Figure 7G). For renal function, the serum levels of BUN, CR, and UA were significantly increased in the HDF group compared with the control group. However, a significant decrease was observed in three dose groups of CCT compared with the HDF group (Figures 7D–F).

### Effects of CCT on Biomarkers of Oxidative Stress

**Figure 8:**
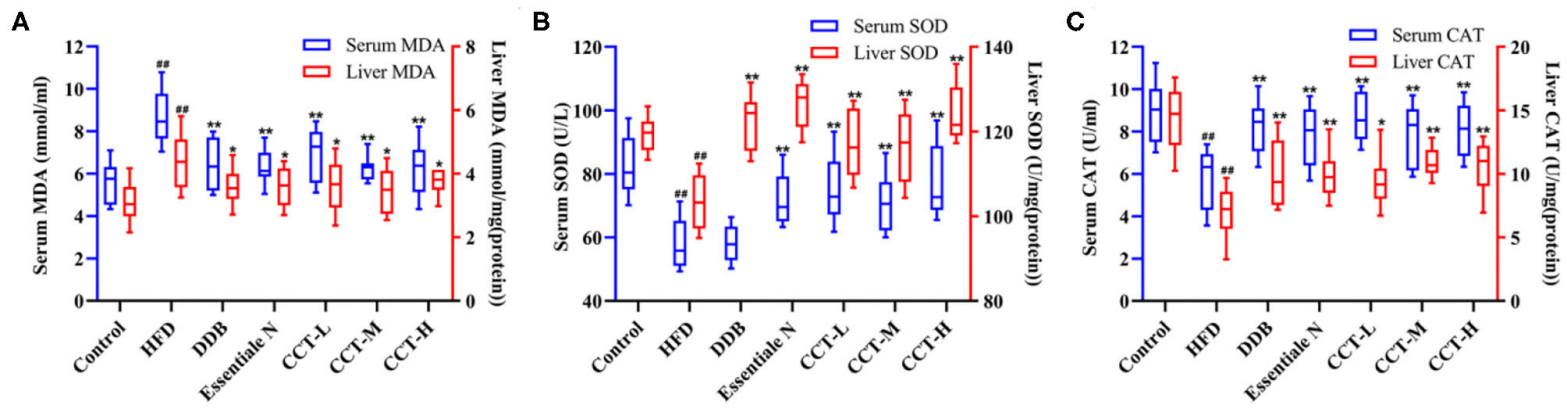
Effects of CCT on oxidative stress biomarkers. **(A)** Effects of CCT on serum MDA and liver TG. **(B)** Effects of CCT on serum SOD and liver SOD. **(C**) Effects of CCT on serum CAT and liver CAT.^##^*P* < 0.01 vs. Control group; *P < 0.05, **P < 0.01 vs. HFD group.

As shown in Figure 8, the serum and liver tissue MDA levels were significantly increased in the HDF group compared with the control group, and there was a significant decrease in the three-dose groups of CCT and DDB group compared with the HDF group (Figure 8A). Similar trends were observed in the activities of SOD and CAT indicating that CCT can alleviate oxidative stress induced by NAFLD (Figures 8B,C).

### Effects of CCT on Biomarkers of Inflammation

**Figure 9:**
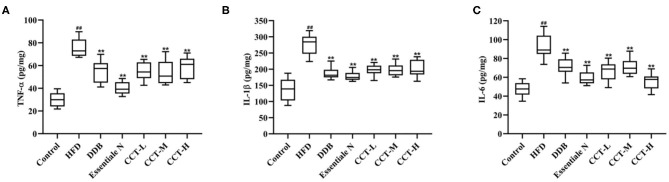
Effects of CCT on inflammation biomarkers. **(A)** Effects of CCT on TNF-α. **(B)** Effects of CCT on IL-1β. **(C)** Effects of CCT on IL-6. ^##^*P* < 0.01 vs. Control group; **P < 0.01 vs. HFD group.

To determine whether CCT could inhibit inflammation in an HFD-induced NAFLD-mouse model, the levels of TNF-α, IL-1β, and IL-6 were measured in the liver tissue. As shown in Figure 9, TNF-α, IL-1β, and IL-6 levels were significantly increased in the HDF group compared with the control group, but significantly decreased in CCT groups, the DDB group and Essential N group.

### Effects of CCT on Liver Histopathology

**Figure 10:**
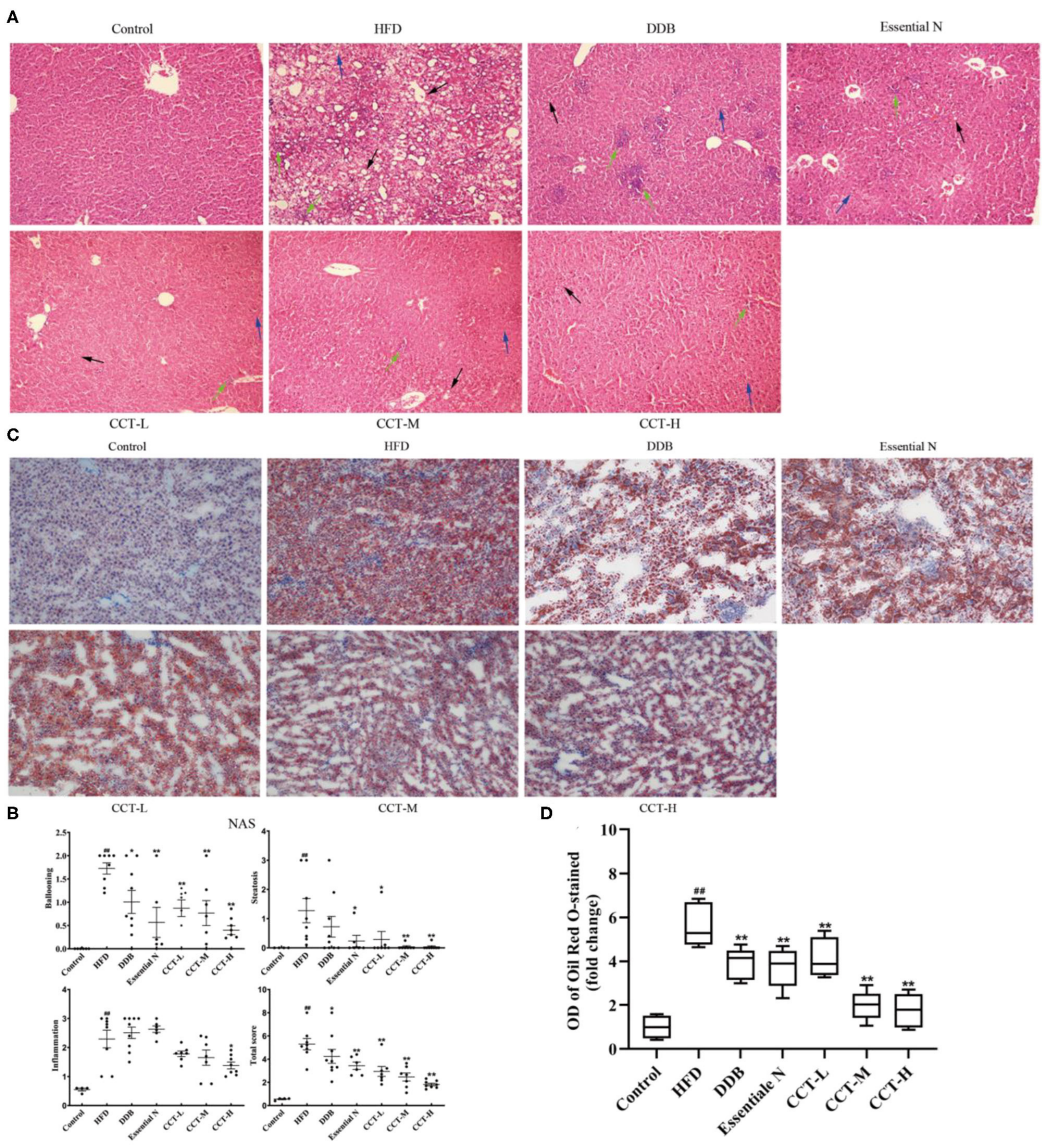
Effects of CCT on liver histopathology. **(A)** Representative photomicrographs of HE staining (×200). The blue arrow represents steatosis, the green arrow represents lobular inflammation, the black arrow represents ballooning degeneration. **(B)** The NAS scores. **(C)** Representative photomicrographs of Oil Red O staining (×200). **(D)** The OD of Oil Red O staining(fold change). ^##^*P* < 0.01 vs. Control group; *P < 0.05, **P < 0.01 vs. HFD group.

In the present study, 10 photomicrographs of HE-staining were selected for use in calculating the NAS score at a magnification of ×200. The NAS score in the HFD group was significantly increased compared with the control group and decreased after CCT treatment. This indicated that CCT significantly attenuated inflammation, steatosis, and ballooning (Figures 10A,B). Besides, Oil Red O staining (Figures 10C,D) showed increased accumulation of lipid droplets in the liver of the HFD group, however, CCT treatment led to a significant decrease in the lipid droplets in the liver.

### Effects of CCT on Nrf2 and HO-1

**Figure 11:**
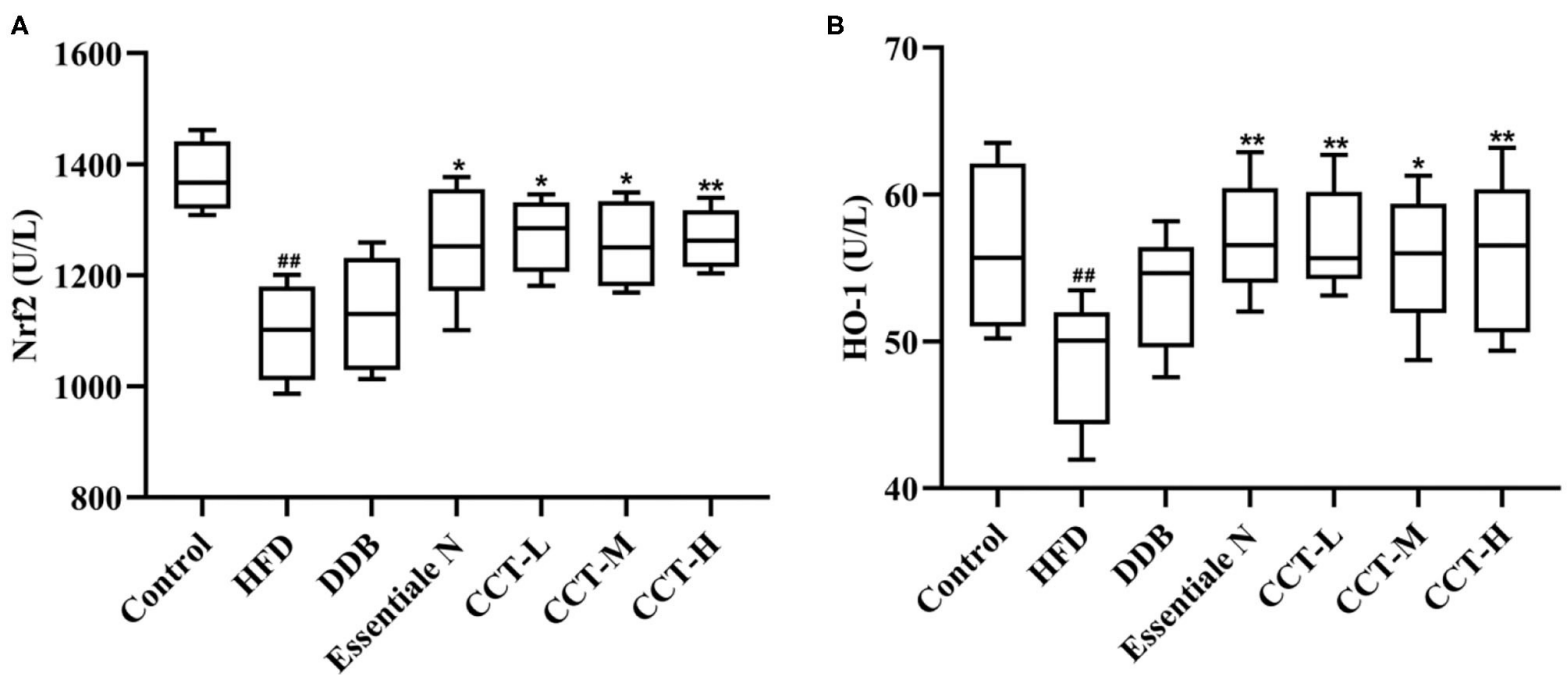
Effects of CCT on Nrf2 and HO-1. **(A)** Effects of CCT on Nrf2. **(B)** Effects of CCT on HO-1. ^##^*P* < 0.01 vs. Control group; *P < 0.05, **P < 0.01 vs. HFD group.

As shown in Figure 11, the expression of Nrf2 and HO-1 significantly decreased in the HDF group compared with the control group. After CCT treatment, the expression of Nrf2 and HO-1 significantly increased in all the groups, except for the DDB group compared with the HDF group.

## Discussion

Network pharmacology is a newly developing interdisciplinary science based on biology, chemical informatics, and bioinformatics, and is also an effective tool used to explore bioactive ingredients from complex components (38). In recent years, network pharmacology has been widely used in traditional Chinese medicine to investigate the potential therapeutic targets and pharmacological mechanisms in different diseases, however, very few studies have used network pharmacology to study the pharmacological effects of saffron. Consistent with our results, a comparative study of the anti-thrombotic effects of saffron and carthami flos based on network pharmacology indicated that CCT was the key bioactive ingredient of saffron (39). Two other studies on systems pharmacology of saffron emphasized the importance of CCT (40, 41). CCT has various biological activities such as anti-tumor, neuroprotective, cardioprotective, hepatoprotective, antidepressant, and improves asthma, Alzheimer's, and diabetes mellitus, etc. (42). In studies of liver diseases, CCT showed comparative beneficial effects on CCL_4_-induced and dengue virus-induced liver damage via induction of antioxidant defense (43, 44). In this study, the therapeutic targets of crocin I, crocin II, and CCT were combined, because orally administered crocins are hydrolyzed to CCT before they are incorporated into the blood circulation, and these findings have been confirmed in several pharmacokinetic studies (45–47). As noted above, CCT was reasonable to reckon to be the critical bioactive ingredient of saffron and further used in the verification experiment.

Although several decades of NAFLD research have resulted in major scientific advancements in pathogenesis and therapeutic target, effective drugs are currently under clinical development (48). The main challenge is that the efficacy of a single target is limited because the pathogenesis and progression of NAFLD involve multiple pathogenic pathways, such as insulin resistance, lipotoxicity, oxidative stress, altered immune/cytokine/mitochondrial functioning, and apoptosis (48–50). In the current study, the possible signaling pathways of saffron intervention in NAFLD were analyzed according to the results obtained from GO, KEGG, and PPI analysis. The results of GO enrichment indicated that saffron may interfere with oxidative stress and nuclear receptor activity in the treatment of NAFLD. The results of KEGG enrichment indicated multiple signaling pathways, including AGE-RAGE signaling pathway in diabetic complications, HIF−1, TNF, adipocytokine, NF-kappa B, PPAR, and other diabetes-related signaling pathways involved in the treatment of NAFLD by using saffron. However, the PPI analysis gave very different results, incicating that AKT1, MAPK1, STAT3, PIK3CA, PIK3R1, RELA, TNF, APP, and JUN were the top 10 core targets. In view of the inconsistent results from KEGG enrichment and PPI, it is necessary to systematically analyze the targets and signaling pathways of saffron in the treatment of NAFLD. Taking into consideration that the results of oxidative stress and nuclear receptor activity from GO enrichment, the 206 common targets were reevaluated, of which NFE2L2 (Nrf2) and HMOX1 (HO-1) have attracted considerable attention. Nrf2 is a tran-scription factor that regulates the expression of several anti-oxidant genes. Previous studies show that Nrf2/HO-1-antioxidant response element (ARE)-antioxidant enzyme play a central mechanistic role in the regulation of the complex antioxidant system in the human body and are key nodes in multiple signaling pathways, including PI3K/AKT/MAPK and IL-6/JAK/STAT3 (51–54). Under normal physiological condition, Kelch-like-ECH-associated protein 1 (Keap1), an Nrf2 repressor, binds to Nrf2 to form a complex and resides in the cytoplasm. However, under stimulation with oxidative and electrophilic chemical signals, Nrf2 is released from Keap1 and transferred to the nucleus, where it binds to the ARE. Here, Keap1 plays an important role in cellular responses to chemical and oxidative stress by regulating the stability and nuclear translocation of Nrf2 protein (55, 56). As one of the key target genes of nuclear Nrf2, HO-1 is a downstream antioxidant enzyme and has a broad cytoprotective effect in various diseases (57). It can be inferred from the above results that CCT may play a role in the treatment of NAFLD by co-intervening the expression of Nrf2 and HO-1. The evidences in biomedical literature in support of this hypothesis have been identified. The antioxidant effect of scutellarin on NAFLD is dependent on PI3K/AKT activation with subsequent Nrf2 nuclear translocation, which increases the expression of HO-1 (58). Resolvin D1 mitigates non-alcoholic steatohepatitis by suppressing the TLR4-MyD88-mediated NF-kappa B and MAPK pathways and activates the Nrf2 pathway in mice (59). In view of the above considerations, Nrf2 and HO-1 were identified as key target genes of the possible signaling pathway and further validated *in vivo* in the current study.

In the validation experiment, HDF was used to induce NAFLD mice to further ascertain whether CCT can ameliorate NAFLD. The results showed that CCT regulates the levels (activities) of TC, TG ALT, AST, and liver index. In the diagnosis of NAFLD, pathological biopsy remains the “golden standard” (60). H&E stained liver sections revealed that CCT can ameliorate hepatocyte ballooning, steatosis, and inflammation to prevent NAFLD. Lipid droplets in the liver were stained using Oil red O, and CCT decreased the lipid droplets. Recent studies have found that NAFLD not only leads to abnormal liver function but also causes extrahepatic disease, such as renal damage (61, 62). Liver steatosis alters the gut barrier function and microbial composition to accumulate toxic metabolites resulting in renal damage (63, 64). In this study, the serum levels of BUN, CR, and UA were analyzed to determine whether CCT can ameliorate renal function caused by NAFLD. The results showed that feeding mice with HFD significantly increased the levels of BUN, CR, and UA, which is consistent with previous clinical trials (65). After CCT treatment, BUN, CR, and UA significantly decreased which was associated with efficient glomerular filtration function and improved NAFLD (66). MDA, a product of lipid oxidation, is used as a marker of oxidative stress. SOD and CAT are enzymatic antioxidant systems, which play important roles in protection against the deleterious effects of hydrogen peroxide and lipid peroxidation in diseases related to oxidative stress (67, 68). In this study, a significant increase in SOD and CAT activities, accompanied by a decrease in MDA level was observed after CCT treatment. These results indicate that CCT can reduce the levels of oxidative stress in the HFD-induced NAFLD model. There is overwhelming evidence that the release of adipocytokines like TNF-α, IL-1β, and IL-6 result in hepatocytes suffering a characteristic variation in their structure developing lobular inflammation and balloon degeneration associated with different degrees of scarring or fibrosis (69). In this study, CCT was found to significantly reduce the levels of TNF-α, IL-1β, and IL-6 which exerts a beneficial effect on NAFLD. After CCT treatment, Nrf2 and HO-1 expression was significantly increased indicating that CCT may activate multiple anti-oxidative signaling pathways to protect against liver lipid peroxidation and hepatitis.

## Conclusion

In conclusion, this study explored, for the first time, the bioactive ingredients of saffron and their therapeutic targets in NAFLD using network pharmacology and animal experiments. CCT was identified as the bioactive compound that ameliorates HFD-induced NAFLD by decreasing level of antioxidants and proinflammatory factors. This study provides a scientific basis for further analysis of the clinical application of CCT in NAFLD.

## Data Availability Statement

The raw data supporting the conclusions of this article will be made available by the authors, without undue reservation, to any qualified researcher.

## Ethics Statement

All experiments were conducted at the Animal experimental research Center of Zhejiang University of Technology. The study was approved by the Animal Protection Research Ethics Committee of Zhejiang University of Technology (20190603064).

## Author Contributions

PW and YT supervised the project, conceived, and designed the research methods. ZX, SL, QL, and YY performed the experiments. YJ and PF performed network pharmacology analysis. ZX wrote the manuscript. YC revised the manuscript. All authors reviewed and approved the final manuscript.

## Conflict of Interest

The authors declare that the research was conducted in the absence of any commercial or financial relationships that could be construed as a potential conflict of interest.

## Abbreviations

ALT: alanine transaminase
AST: aspartate aminotransferase
BUN: blood urea nitrogen
CAT: catalase
CCT: crocetin
CR: creatinine
DL: drug-likeness
DAVID: visualization and Integrated Discovery
GO: Gene ontology
H&E: haematoxylin and eosin staining
HFD: high-fat diet
HO-1: hemeoxygenase-1
IL-1β: interleukin-1β
IL-6: interleukin-6
KEGG: Kyoto Encyclopedia of Genes and Genomes
MDA: malondialdehyde
NAFLD: non-alcoholic fatty liver disease
NAS: NAFLD activity score
Nrf2: nuclear factor erythroid-related factor
OB: oral bioavailability
PPI: protein-protein interaction network
SOD: superoxide dismutase
TC: total cholesterol
TCMSP: traditional Chinese Medicine Systems Pharmacology
TCMID: traditional Chinese Medicine Integrated Database
TG: triglyceride
TNF-α: tumor necrosis factor-α
UA: uric acid.
