# Supplementary material for: Exploring the Protective Effects and Mechanism of Crocetin From Saffron Against NAFLD by Network Pharmacology and Experimental Validation
Source: Front Med (Lausanne). 2021 Jun 9;8:681391. doi: 10.3389/fmed.2021.681391 (PMC8219931; doi:10.3389/fmed.2021.681391)
Supplement: Supplementary file 1 [file Data_Sheet_1.pdf]

Supplementary Material

Supplement Table S1. More detailed information of 206 common targets.

Uniprot ID	Target gene	Target protein	Uniprot ID	Target gene	Target protein
P19320	VCAM1	Vascular cell adhesion protein 1	Q01959	SLC6A3	Solute carrier family 6 member 3
P23219	PTGS1	Prostaglandin G/H synthase 1	P78527	PRKDC	DNA-dependent protein kinase catalytic subunit
P10275	AR	Androgen receptor	P42345	MTOR	Serine/threonine-protein kinase mTOR
P35354	PTGS2	Prostaglandin G/H synthase 2	Q96IY4	CPB2	Carboxypeptidase B2
Q04206	RELA	Transcription factor p65	P30556	AGTR1	Type-1 angiotensin II receptor
P00533	EGFR	Epidermal growth factor receptor	P24863	CCNC	Cyclin-C
P31749	AKT1	RAC-alpha serine/threonine-protein kinase	P49336	CDK8	Cyclin-dependent kinase 8
P15692	VEGFA	Vascular endothelial growth factor A	P05556	ITGB1	Integrin beta-1
P24385	CCND1	G1/S-specific cyclin-D1	P08581	MET	Hepatocyte growth factor receptor
P10415	BCL2	Apoptosis regulator Bcl-2	Q96RR4	CAMKK2	Calcium/calmodulin-dependent protein kinase kinase 2
P01100	FOS	Proto-oncogene c-Fos	Q9UHD2	TBK1	TANK-binding kinase 1
P38936	CDKN1A	Cyclin-dependent kinase inhibitor 1	Q13822	ENPP2	Ectonucleotide pyrophosphatase/phosphodiesterase family member 2
Q07812	BAX	Apoptosis regulator BAX	P15085	CPA1	Carboxypeptidase A1
P55211	CASP9	Caspase-9	P25025	CXCR2	C-X-C chemokine receptor type 2
P08253	MMP2	Matrix metalloproteinase-2	P47871	GCGR	Glucagon receptor
P14780	MMP9	Matrix metalloproteinase-9	P05023	ATP1A1	Sodium/potassium-transporting ATPase subunit alpha-1
P28482	MAPK1	Mitogen-activated protein kinase 1	P40763	STAT3	Signal transducer and activator of transcription 3
P22301	IL10	Interleukin-10	Q05655	PRKCD	Protein kinase C delta type
P01133	EGF	Pro-epidermal growth factor	P11473	VDR	Vitamin D3 receptor
P05412	JUN	Transcription factor AP-1	P18031	PTPN1	Tyrosine-protein phosphatase non-receptor type 1
P05231	IL6	Interleukin-6	Q15257	PTPA	Serine/threonine-protein phosphatase 2A activator
O95433	AHSA1	Activator of 90 kDa heat shock protein ATPase homolog 1	Q15393	SF3B3	Splicing factor 3B subunit 3

P42574	CASP3	Caspase-3	P00734	F2	Activation peptide fragment 2
Q9H3D4	TP63	Tumor protein 63	Q02156	PRKCE	Protein kinase C epsilon type
Q14790	CASP8	Caspase-8	P28845	HSD11B1	Corticosteroid 11-beta-dehydrogenase isozyme 1
P00441	SOD1	Superoxide dismutase 1	P05230	FGF1	Fibroblast growth factor 1
P17252	PRKCA	Protein kinase C alpha type	Q9Y251	HPSE	Heparanase
P03956	MMP1	Matrix metalloproteinase-1	P00915	CA1	Carbonic anhydrase 1
Q16665	HIF1A	Hypoxia-inducible factor 1-alpha	P14416	DRD2	Dopamine D2 receptor
P42224	STAT1	Signal transducer and activator of transcription 1	P28223	HTR2A	5-hydroxytryptamine receptor 2A
P37231	PPARG	Peroxisome proliferator-activated receptor gamma	P10635	CYP2D6	Cytochrome P450 2D6
Q13085	ACACA	Acetyl-CoA carboxylase 1	P28222	HTR1B	5-hydroxytryptamine receptor 1B
P09601	HMOX1	Heme oxygenase 1	P35372	OPRM1	Mu-type opioid receptor
P08684	CYP3A4	Cytochrome P450 3A4	P37268	FDFT1	Squalene synthase
P05177	CYP1A2	Cytochrome P450 1A2	Q14534	SQLE	Squalene monooxygenase
Q03135	CAV1	Caveolin-1	P17931	LGALS3	Galectin-3
P01106	MYC	Myc proto-oncogene protein	P11166	SLC2A1	Solute carrier family 2
P13726	F3	Tissue factor	O95342	ABCB11	ATP-binding cassette sub-family B member 11
P04798	CYP1A1	Cytochrome P450 1A1	P34972	CNR2	Cannabinoid receptor 2
P05362	ICAM1	Intercellular adhesion molecule 1	P55085	F2RL1	Proteinase-activated receptor 2
P01584	IL1B	Interleukin-1 beta	P11717	IGF2R	Cation-independent mannose-6-phosphate receptor
P13500	CCL2	C-C motif chemokine 2	P23975	SLC6A2	Sodium-dependent noradrenaline transporter
P10145	CXCL8	Interleukin-8	Q9NR96	TLR9	Toll-like receptor 9
P05771	PRKCB	Protein kinase C beta type	P20309	CHRM3	Muscarinic acetylcholine receptor M3
Q9NRD8	DUOX2	Dual oxidase 2	P31639	SLC5A2	Sodium/glucose cotransporter 2
P29474	NOS3	Nitric oxide synthase, endothelial	Q9NPH5	NOX4	NADPH oxidase 4
P04792	HSPB1	Heat shock protein beta-1	P47989	XDH	Xanthine dehydrogenase/oxidase
P49888	SULT1E1	Sulfotransferase 1E1	P21397	MAOA	Monoamine oxidase type A
P60568	IL2	Interleukin-2	P08069	IGF1R	Insulin-like growth factor 1 receptor
O75469	NR1I2	Nuclear receptor subfamily 1 group I member 2	P11511	CYP19A1	Cytochrome P450 19A1
P00797	REN	Renin	P14174	MIF	Macrophage migration inhibitory factor
Q13526	PIN1	Peptidyl-prolyl cis-trans isomerase NIMA-interacting 1	Q99523	SORT1	Sortilin

P05121	SERPINE1	Plasminogen activator inhibitor 1	P21917	DRD4	D(4) dopamine receptor
P02452	COL1A1	Collagen alpha-1(I) chain	P27986	PIK3R1	Phosphatidylinositol 3-kinase regulatory subunit alpha
P01579	IFNG	Interferon gamma	P45452	MMP13	Matrix metalloproteinase-13
P09917	ALOX5	Polyunsaturated fatty acid 5-lipoxygenase	P16050	ALOX15	Polyunsaturated fatty acid lipoxygenase ALOX15
P01583	IL1A	Interleukin-1 alpha	P18054	ALOX12	Polyunsaturated fatty acid lipoxygenase ALOX12
P05164	MPO	Myeloperoxidase	Q9UM73	ALK	Anaplastic lymphoma kinase
P14598	NCF1	Neutrophil cytosol factor 1	P10636	MAPT	Microtubule-associated protein tau
P09211	GSTP1	Glutathione S-transferase P	P22303	ACHE	Acetylcholinesterase
Q16236	NFE2L2	Nuclear factor erythroid 2-related factor 2	P48736	PIK3CG	Phosphatidylinositol 4,5-bisphosphate 3-kinase catalytic subunit gamma isoform
P15559	NQO1	NAD(P)H dehydrogenase	P11474	ESRRA	Steroid hormone receptor ERR1
P09874	PARP1	Poly [ADP-ribose] polymerase 1	P05067	APP	Amyloid-beta precursor protein
P35869	AHR	Aryl hydrocarbon receptor	P02766	TTR	Transthyretin
Q9NR83	SLC2A4	SLC2A4 regulator	O60218	AKR1B10	Aldo-keto reductase family 1 member B10
P02461	COL3A1	Collagen alpha-1(III) chain	O14746	TERT	Telomerase reverse transcriptase
Q14994	NR1I3	Nuclear receptor subfamily 1 group I member 3	Q20BH0	CFTR	Cystic fibrosis transmembrane conductance regulator
P06213	INSR	Insulin receptor	P06276	BCHE	Butyrylcholine esterase
Q07869	PPARA	Peroxisome proliferator-activated receptor alpha	P00747	PLG	Plasminogen
Q03181	PPARD	Peroxisome proliferator-activated receptor delta	P23141	CES1	Liver carboxylesterase 1
P02741	CRP	C-reactive protein	P34949	MPI	Mannose-6-phosphate isomerase
P02778	CXCL10	C-X-C motif chemokine 10	O00748	CES2	Cocaine esterase
O15111	CHUK	Inhibitor of nuclear factor kappa-B kinase subunit alpha	P07327	ADH1A	Alcohol dehydrogenase 1A
P10451	SPP1	Secreted phosphoprotein 1	P00326	ADH1C	Alcohol dehydrogenase 1C
Q13950	RUNX2	Q13950	P00325	ADH1B	Alcohol dehydrogenase 1B
Q01094	E2F1	Transcription factor E2F1	P34969	HTR7	5-hydroxytryptamine receptor 7, 5-HT-7
P07339	CTSD	Cathepsin D	P10276	RARA	Retinoic acid receptor alpha
P17936	IGFBP3	Insulin-like growth factor-binding protein 3	Q99685	MGLL	Monoglyceride lipase
P11717	IGF2	Cation-independent mannose-6-phosphate receptor	P08319	ADH4	Alcohol dehydrogenase 4

P29965	CD40LG	CD40 ligand	P04150	NR3C1	Nuclear receptor subfamily 3 group C member 1
P27169	PON1	Serum paraoxonase/arylesterase 1	P07858	CTSB	Cathepsin B
P09488	GSTM1	Glutathione S-transferase Mu 1	P48443	RXRG	Retinoic acid receptor RXR-gamma
P28161	GSTM2	Glutathione S-transferase Mu 2	P19793	RXRA	Retinoic acid receptor RXR-alpha
P03973	SLPI	Antileukoproteinase	Q8IXJ6	SIRT2	NAD-dependent protein deacetylase sirtuin-2
O14920	IKBKB	Inhibitor of nuclear factor kappa-B kinase subunit beta	Q99572	P2RX7	P2X purinoceptor 7
P45983	MAPK8	Mitogen-activated protein kinase 8	P07202	TPO	Thyroid peroxidase
P35228	NOS2	Nitric oxide synthase	P21757	MSR1	Macrophage scavenger receptor types I and II
P78380	OLR1	Oxidized low-density lipoprotein receptor 1	P11940	PABPC1	Polyadenylate-binding protein 1
P49841	GSK3B	Glycogen synthase kinase-3 beta	P11509	CYP2A6	Cytochrome P450 2A6
P12821	ACE	Angiotensin-converting enzyme	Q9BY41	HDAC8	Histone deacetylase 8
P15121	AKR1B1	Aldo-keto reductase family 1 member B1	P01375	TNF	Tumor necrosis factor
P14550	AKR1A1	Aldo-keto reductase family 1 member A1	P25774	CTSS	Cathepsin S
P31645	SLC6A4	Solute carrier family 6 member 4	P08235	NR3C2	Mineralocorticoid receptor
P07900	HSP90AA1	Heat shock protein HSP 90-alpha	P04278	SHBG	Sex hormone-binding globulin
P42336	PIK3CA	Phosphoinositide-3-kinase catalytic alpha polypeptide	P07148	FABP1	Fatty acid-binding protein
O75907	DGAT1	Diacylglycerol O-acyltransferase 1	Q8NER1	TRPV1	Transient receptor potential cation channel subfamily V member 1
P27338	MAOB	Monoamine oxidase type B	P11142	HSPA8	Heat shock cognate 71 kDa protein
P15090	FABP4	Fatty acid-binding protein 4	P11021	HSPA5	Heat shock protein 70 family protein 5
P22736	NR4A1	Nuclear receptor subfamily 4 group A member 1	P16278	GLB1	Beta-galactosidase
P03372	ESR1	Estrogen receptor	P23526	AHCY	Adenosylhomocysteinase
Q16875	PFKFB3	6-phosphofructo-2-kinase/fructose-2,6-bisphosphatase 3	P78536	ADAM17	Disintegrin and metalloproteinase domain-containing protein 17
P0DMV8	HSPA1A	Heat shock 70 kDa protein 1A	P17948	FLT1	Vascular endothelial growth factor receptor 1
P50416	CPT1A	Carnitine O-palmitoyltransferase 1	O75460	ERN1	Endoplasmic reticulum-to-nucleus signaling 1

Supplement Table S2. The specific targets in signaling pathways.

Signaling pathway	Specific targets
NON-ALCOHOLIC FATTY LIVER DISEASE	IL-6, TNF-α, NF-κB, INSR, RXR, PI3K, AKT, GSK-3, PPAR-α, JNK1/2, IRE1-α, CASP8, Bax, JNK1, IKK-β, AP-1, c-Jun, IL-1, IL-8. CASP3.
AGE-RAGE SIGNALING PATHWAY IN DIABETIC COMPLICATIONS	AT1R, ANDPH, PKC, PI3K, ERK1/2, JNK, AKT, NOS, STAT3, STAT1, PKCβII, AP-1, NF-κB, Bcl-2, Bax, COL, VEGF, MCP-1, PAI-1, VCAM-1, ICAM-1, MMP2, IL-1, IL-8, IL-6, TNF-α, TF, CASP3, COLl, CycD1.
TNF SIGNALING PATHWAY	TNF, NF-κB, IKKβ, IKKα, CASP8, CASP3, JNK1/2, ERK1/2, AP-1, PI3K, AKT, IKKs, JNK, c-Jun, Ccl2, CXCL10, IL1B, IL-6, Fos, Jun, MMP9, ICAM1, VCAM1, PTGS2.
HIF-1 SIGNALING PATHWAY	IL-6, PI3K, AKT, mTOR, PKC, HIF-1α, ERK, PKC, STAT3, NF-κB, VEGF, FLT1, EGF, PAI-1, iNOS, eNOS, HMOX1, Glut1, Bcl-2.

Supplement Table S3. Details of the PPI network.

#node1	node2	node1_string_id	node2_string_id	neighborhood_on_chromosome	gene_fusion	phylogenetic_cooccurrence	homology	coexpression	experimentally_determined_interaction	database_annotated	automated_textmining	combined_score
ACE	REN	9606.ENSP00000290866	9606.ENSP00000272190	0	0	0	0	0	0	0	0.959	0.959
ACE	AGTR1	9606.ENSP00000290866	9606.ENSP00000419422	0	0	0	0	0.06	0	0	0.927	0.928
ADAM17	SORT1	9606.ENSP00000309968	9606.ENSP00000256637	0	0	0	0	0	0	0.9	0.209	0.917
ADAM17	EGF	9606.ENSP00000309968	9606.ENSP00000265171	0	0	0	0	0	0	0.9	0.806	0.979
ADAM17	PRKCB	9606.ENSP00000309968	9606.ENSP00000305355	0	0	0	0	0	0	0.9	0	0.900
ADAM17	PRKCA	9606.ENSP00000309968	9606.ENSP00000408695	0	0	0	0	0	0	0.9	0.083	0.904
ADAM17	TNF	9606.ENSP00000309968	9606.ENSP00000398698	0	0	0	0	0.061	0.379	0.9	0.692	0.979
ADH1A	ADH1C	9606.ENSP00000209668	9606.ENSP00000426083	0	0	0.449	0.985	0.182	0	0.9	0.86	0.916
ADH1A	ADH1B	9606.ENSP00000209668	9606.ENSP00000306606	0	0	0.449	0.985	0.828	0	0.9	0.892	0.982
ADH1B	MAOA	9606.ENSP00000306606	9606.ENSP00000340684	0	0	0	0	0.103	0	0.9	0.368	0.938
ADH1B	ADH1C	9606.ENSP00000306606	9606.ENSP00000426083	0	0	0.449	0.986	0.179	0.692	0.9	0.921	0.973
ADH4	MAOA	9606.ENSP00000265512	9606.ENSP00000340684	0	0	0	0	0.061	0	0.9	0.203	0.918
AGTR1	CFTR	9606.ENSP00000419422	9606.ENSP00000003084	0	0	0	0	0	0	0.9	0.089	0.905
AGTR1	CHRM3	9606.ENSP00000419422	9606.ENSP00000255380	0	0	0	0.609	0	0	0.9	0.349	0.912
AGTR1	PIK3CA	9606.ENSP00000419422	9606.ENSP00000263967	0	0	0	0	0	0.05	0.9	0.053	0.902
AGTR1	EGF	9606.ENSP00000419422	9606.ENSP00000265171	0	0	0	0	0	0.062	0.9	0.4	0.938
AGTR1	REN	9606.ENSP00000419422	9606.ENSP00000272190	0	0	0	0	0.055	0	0	0.954	0.955
AGTR1	EGFR	9606.ENSP00000419422	9606.ENSP00000275493	0	0	0	0	0.076	0.406	0.9	0.424	0.964
AGTR1	APP	9606.ENSP00000419422	9606.ENSP00000284981	0	0	0	0	0	0	0.9	0.221	0.918
AGTR1	F2RL1	9606.ENSP00000419422	9606.ENSP00000296677	0	0	0	0.693	0	0	0.9	0.195	0.904

AGTR1	CXCL8	9606.ENSP00000419422	9606.ENSP00000306512	0	0	0	0	0	0	0.9	0.293	0.926
AGTR1	F2	9606.ENSP00000419422	9606.ENSP00000308541	0	0	0	0	0.063	0	0.9	0.325	0.931
AGTR1	IGF2R	9606.ENSP00000419422	9606.ENSP00000349437	0	0	0	0	0	0	0.9	0.257	0.922
AGTR1	GCGR	9606.ENSP00000419422	9606.ENSP00000383558	0	0	0	0	0.064	0	0.9	0.189	0.917
AGTR1	OPRM1	9606.ENSP00000419422	9606.ENSP00000394624	0	0	0	0.753	0	0	0.9	0.268	0.905
AGTR1	HTR2A	9606.ENSP00000419422	9606.ENSP00000437737	0	0	0	0.587	0	0	0.9	0.187	0.906
AGTR1	PIK3R1	9606.ENSP00000419422	9606.ENSP00000428056	0	0	0	0	0	0.057	0.9	0.099	0.907
AGTR1	HSPA8	9606.ENSP00000419422	9606.ENSP00000432083	0	0	0	0	0	0.053	0.9	0.185	0.916
AHR	HSP90AA1	9606.ENSP00000242057	9606.ENSP00000335153	0	0	0	0	0	0.472	0.9	0.818	0.989
AHR	CYP1A1	9606.ENSP00000242057	9606.ENSP00000369050	0	0	0	0	0.061	0	0.9	0.975	0.997
AHSA1	HSP90AA1	9606.ENSP00000216479	9606.ENSP00000335153	0	0	0	0	0.689	0.827	0	0.934	0.996
AKR1A1	MGLL	9606.ENSP00000361140	9606.ENSP00000265052	0.045	0	0	0	0.063	0	0.9	0	0.902
AKR1A1	MAOB	9606.ENSP00000361140	9606.ENSP00000367309	0	0	0	0	0.062	0	0.9	0.223	0.92
AKR1B1	MGLL	9606.ENSP00000285930	9606.ENSP00000265052	0.045	0	0	0	0.063	0	0.9	0	0.902
AKR1B1	GLB1	9606.ENSP00000285930	9606.ENSP00000306920	0.058	0	0	0	0.055	0	0.9	0.085	0.907
AKR1B1	AKR1B10	9606.ENSP00000285930	9606.ENSP00000352584	0	0	0.449	0.975	0.099	0	0.9	0.743	0.908
AKR1B10	MGLL	9606.ENSP00000352584	9606.ENSP00000265052	0.045	0	0	0	0.063	0	0.9	0.058	0.904
AKR1B10	GLB1	9606.ENSP00000352584	9606.ENSP00000306920	0.058	0	0	0	0.055	0	0.9	0.063	0.905
AKT1	MAPK1	9606.ENSP00000451828	9606.ENSP00000215832	0	0	0.304	0.592	0.066	0.146	0.8	0.948	0.905
AKT1	IL2	9606.ENSP00000451828	9606.ENSP00000226730	0	0	0	0	0	0	0.9	0.793	0.978
AKT1	CCND1	9606.ENSP00000451828	9606.ENSP00000227507	0	0	0	0	0.061	0	0	0.941	0.943
AKT1	HSPB1	9606.ENSP00000451828	9606.ENSP00000248553	0	0	0	0	0	0.698	0	0.864	0.957
AKT1	RARA	9606.ENSP00000451828	9606.ENSP00000254066	0	0	0	0	0.088	0.407	0.9	0.325	0.958
AKT1	PIK3CA	9606.ENSP00000451828	9606.ENSP00000263967	0	0	0	0	0.063	0.468	0.9	0.978	0.998
AKT1	STAT3	9606.ENSP00000451828	9606.ENSP00000264657	0	0	0	0	0	0.124	0.9	0.938	0.994
AKT1	EGF	9606.ENSP00000451828	9606.ENSP00000265171	0	0	0	0	0	0.05	0	0.932	0.933

AKT1	SOD1	9606.ENSP00000451828	9606.ENSP00000270142	0	0	0	0	0	0.064	0.9	0.64	0.963
AKT1	NOS3	9606.ENSP00000451828	9606.ENSP00000297494	0	0	0	0	0.049	0.693	0.9	0.97	0.999
AKT1	INSR	9606.ENSP00000451828	9606.ENSP00000303830	0	0	0	0.559	0.052	0.288	0.9	0.677	0.948
AKT1	TERT	9606.ENSP00000451828	9606.ENSP00000309572	0	0	0	0	0.055	0.379	0.9	0.783	0.985
AKT1	CASP3	9606.ENSP00000451828	9606.ENSP00000311032	0	0	0	0	0.055	0.393	0	0.95	0.969
AKT1	PRKDC	9606.ENSP00000451828	9606.ENSP00000313420	0	0	0	0	0.058	0.432	0.9	0.805	0.988
AKT1	SLC2A4	9606.ENSP00000451828	9606.ENSP00000320935	0	0	0	0	0	0.261	0	0.902	0.925
AKT1	GSK3B	9606.ENSP00000451828	9606.ENSP00000324806	0	0	0.218	0.576	0.049	0.995	0.9	0.918	0.999
AKT1	NOS2	9606.ENSP00000451828	9606.ENSP00000327251	0	0	0	0	0.063	0.065	0.8	0.543	0.909
AKT1	CASP9	9606.ENSP00000451828	9606.ENSP00000330237	0	0	0	0	0	0.393	0.9	0.919	0.994
AKT1	HSP90AA1	9606.ENSP00000451828	9606.ENSP00000335153	0	0	0	0	0.061	0.806	0.9	0.92	0.998
AKT1	CAV1	9606.ENSP00000451828	9606.ENSP00000339191	0	0	0	0	0	0.057	0.9	0.815	0.981
AKT1	E2F1	9606.ENSP00000451828	9606.ENSP00000345571	0	0	0	0	0.091	0.124	0.9	0.481	0.953
AKT1	PIK3CG	9606.ENSP00000451828	9606.ENSP00000352121	0	0	0	0	0.063	0.174	0.9	0.771	0.979
AKT1	RXRG	9606.ENSP00000451828	9606.ENSP00000352900	0	0	0	0	0	0.085	0.9	0.079	0.908
AKT1	MTOR	9606.ENSP00000451828	9606.ENSP00000354558	0	0	0	0	0.08	0.872	0.9	0.972	0.999
AKT1	CHUK	9606.ENSP00000451828	9606.ENSP00000359424	0	0	0	0.593	0	0.682	0.9	0.673	0.975
AKT1	CD40LG	9606.ENSP00000451828	9606.ENSP00000359663	0	0	0	0	0	0.072	0.9	0.482	0.947
AKT1	JUN	9606.ENSP00000451828	9606.ENSP00000360266	0	0	0	0	0	0.072	0	0.91	0.913
AKT1	PTPN1	9606.ENSP00000451828	9606.ENSP00000360683	0	0	0	0	0.09	0.397	0.9	0.796	0.987
AKT1	AR	9606.ENSP00000451828	9606.ENSP00000363822	0	0	0	0	0	0.407	0	0.89	0.932
AKT1	BCL2	9606.ENSP00000451828	9606.ENSP00000381185	0	0	0	0	0.061	0	0.9	0.426	0.941
AKT1	CDKN1A	9606.ENSP00000451828	9606.ENSP00000384849	0	0	0	0	0	0.696	0.9	0.851	0.995
AKT1	ESR1	9606.ENSP00000451828	9606.ENSP00000405330	0	0	0	0	0.055	0.407	0.9	0.867	0.991
AKT1	RXRA	9606.ENSP00000451828	9606.ENSP00000419692	0	0	0	0	0.085	0.085	0.9	0.271	0.93
AKT1	PIK3R1	9606.ENSP00000451828	9606.ENSP00000428056	0	0	0	0	0.062	0.447	0.9	0.835	0.99

AKT1	IKBKB	9606.ENSP00000451828	9606.ENSP00000430684	0	0	0	0.601	0	0.682	0.9	0.574	0.974
AKT1	HIF1A	9606.ENSP00000451828	9606.ENSP00000437955	0	0	0	0	0	0.362	0.9	0.827	0.988
AKT1	NR4A1	9606.ENSP00000451828	9606.ENSP00000440864	0	0	0	0	0	0.4	0.9	0.76	0.984
AKT1	VEGFA	9606.ENSP00000451828	9606.ENSP00000478570	0	0	0	0	0.063	0.342	0	0.95	0.966
AKT1	MYC	9606.ENSP00000451828	9606.ENSP00000479618	0	0	0	0	0.052	0.156	0.9	0.915	0.992
ALK	PIK3CA	9606.ENSP00000373700	9606.ENSP00000263967	0	0	0	0	0.058	0.124	0.8	0.736	0.95
ALOX12	ALOX15	9606.ENSP00000251535	9606.ENSP00000458832	0	0	0.407	0.97	0.061	0	0.9	0.709	0.905
ALOX12	ALOX5	9606.ENSP00000251535	9606.ENSP00000363512	0	0	0.386	0.911	0.063	0	0.9	0.863	0.912
ALOX12	PTGS2	9606.ENSP00000251535	9606.ENSP00000356438	0	0	0	0	0.061	0	0.9	0.526	0.951
ALOX12	PTGS1	9606.ENSP00000251535	9606.ENSP00000354612	0	0	0	0	0.107	0	0.9	0.57	0.958
ALOX15	CYP3A4	9606.ENSP00000458832	9606.ENSP00000337915	0	0	0	0	0	0	0.9	0.087	0.904
ALOX15	CYP1A2	9606.ENSP00000458832	9606.ENSP00000342007	0	0	0	0	0	0	0.9	0.057	0.901
ALOX15	PTGS1	9606.ENSP00000458832	9606.ENSP00000354612	0	0	0	0	0.061	0	0.9	0.594	0.958
ALOX15	PTGS2	9606.ENSP00000458832	9606.ENSP00000356438	0	0	0	0	0.061	0	0.9	0.601	0.959
ALOX15	ALOX5	9606.ENSP00000458832	9606.ENSP00000363512	0	0	0.298	0.905	0.062	0	0.9	0.891	0.912
ALOX5	PTGS1	9606.ENSP00000363512	9606.ENSP00000354612	0	0	0	0	0.095	0	0.9	0.728	0.973
ALOX5	PTGS2	9606.ENSP00000363512	9606.ENSP00000356438	0	0	0	0	0.08	0	0.9	0.758	0.975
APP	DRD4	9606.ENSP00000284981	9606.ENSP00000176183	0	0	0	0	0	0	0.9	0.251	0.921
APP	MAPK1	9606.ENSP00000284981	9606.ENSP00000215832	0	0	0	0	0.062	0.08	0.9	0.492	0.95
APP	SERPINE1	9606.ENSP00000284981	9606.ENSP00000223095	0	0	0	0	0	0	0.9	0.25	0.921
APP	TTR	9606.ENSP00000284981	9606.ENSP00000237014	0	0	0	0	0.063	0.379	0.9	0.546	0.97
APP	CHRM3	9606.ENSP00000284981	9606.ENSP00000255380	0	0	0	0	0.081	0	0.9	0.093	0.909
APP	PIK3CA	9606.ENSP00000284981	9606.ENSP00000263967	0	0	0	0	0	0	0.9	0.123	0.908
APP	EGF	9606.ENSP00000284981	9606.ENSP00000265171	0	0	0	0	0.061	0	0.9	0.51	0.949
APP	GCGR	9606.ENSP00000284981	9606.ENSP00000383558	0	0	0	0	0	0	0.9	0.054	0.901
APP	F2RL1	9606.ENSP00000284981	9606.ENSP00000296677	0	0	0	0	0	0	0.9	0.055	0.901

APP	HTR1B	9606.ENSP00000284981	9606.ENSP00000358963	0	0	0	0	0.075	0	0.9	0.079	0.907
APP	IGFBP3	9606.ENSP00000284981	9606.ENSP00000370473	0	0	0	0	0.088	0	0.9	0.146	0.915
APP	PIK3R1	9606.ENSP00000284981	9606.ENSP00000428056	0	0	0	0	0	0	0.9	0.19	0.915
APP	OPRM1	9606.ENSP00000284981	9606.ENSP00000394624	0	0	0	0	0	0	0.9	0.197	0.916
APP	CNR2	9606.ENSP00000284981	9606.ENSP00000363596	0	0	0	0	0	0	0.9	0.195	0.916
APP	CXCR2	9606.ENSP00000284981	9606.ENSP00000319635	0	0	0	0	0	0	0.9	0.211	0.917
APP	SPP1	9606.ENSP00000284981	9606.ENSP00000378517	0	0	0	0	0.076	0	0.9	0.187	0.918
APP	CXCL10	9606.ENSP00000284981	9606.ENSP00000305651	0	0	0	0	0	0	0.9	0.243	0.921
APP	HTR2A	9606.ENSP00000284981	9606.ENSP00000437737	0	0	0	0	0.061	0	0.9	0.237	0.922
APP	IGF2	9606.ENSP00000284981	9606.ENSP00000391826	0	0	0	0	0.066	0	0.9	0.315	0.93
APP	DRD2	9606.ENSP00000284981	9606.ENSP00000354859	0	0	0	0	0.063	0	0.9	0.33	0.931
APP	CXCL8	9606.ENSP00000284981	9606.ENSP00000306512	0	0	0	0	0	0	0.9	0.376	0.934
APP	RELA	9606.ENSP00000284981	9606.ENSP00000384273	0	0	0	0	0	0	0.9	0.408	0.938
APP	PLG	9606.ENSP00000284981	9606.ENSP00000308938	0	0	0	0	0	0.132	0.9	0.384	0.941
APP	VEGFA	9606.ENSP00000284981	9606.ENSP00000478570	0	0	0	0	0.067	0	0.9	0.44	0.943
APP	F2	9606.ENSP00000284981	9606.ENSP00000308541	0	0	0	0	0	0.132	0.9	0.413	0.944
APP	MAPT	9606.ENSP00000284981	9606.ENSP00000340820	0	0	0	0	0.064	0.379	0	0.919	0.949
APP	IL6	9606.ENSP00000284981	9606.ENSP00000385675	0	0	0	0	0	0	0.9	0.589	0.957
APP	CASP8	9606.ENSP00000284981	9606.ENSP00000351273	0	0	0	0	0	0.379	0.9	0.39	0.958
APP	MAPK8	9606.ENSP00000284981	9606.ENSP00000378974	0	0	0	0	0.052	0.294	0.9	0.791	0.984
AR	CCND1	9606.ENSP00000363822	9606.ENSP00000227507	0	0	0	0	0.061	0.384	0.9	0.795	0.986
AR	NR3C1	9606.ENSP00000363822	9606.ENSP00000231509	0	0	0	0.816	0	0.379	0.9	0.806	0.944
AR	PIK3CA	9606.ENSP00000363822	9606.ENSP00000263967	0	0	0	0	0	0	0.9	0.473	0.945
AR	GSK3B	9606.ENSP00000363822	9606.ENSP00000324806	0	0	0	0	0	0.407	0.9	0.49	0.967
AR	HSP90AA1	9606.ENSP00000363822	9606.ENSP00000335153	0	0	0	0	0	0.474	0.9	0.803	0.988
AR	NR3C2	9606.ENSP00000363822	9606.ENSP00000350815	0	0	0	0.779	0.061	0.379	0.9	0.67	0.945

AR	CASP8	9606.ENSP00000363822	9606.ENSP00000351273	0	0	0	0	0	0.472	0.9	0.399	0.965
AR	HSPA8	9606.ENSP00000363822	9606.ENSP00000432083	0	0	0	0	0	0.109	0.9	0.348	0.936
AR	MAPK8	9606.ENSP00000363822	9606.ENSP00000378974	0	0	0	0	0	0	0.9	0.479	0.945
AR	PIK3R1	9606.ENSP00000363822	9606.ENSP00000428056	0	0	0	0	0	0.153	0.9	0.517	0.955
AR	HSPA1A	9606.ENSP00000363822	9606.ENSP00000364802	0	0	0	0	0	0.423	0.9	0.61	0.975
BAX	TP63	9606.ENSP00000293288	9606.ENSP00000264731	0	0	0	0	0	0	0.9	0.056	0.901
BAX	MAPK8	9606.ENSP00000293288	9606.ENSP00000378974	0	0	0	0	0	0.053	0.9	0.525	0.951
BAX	BCL2	9606.ENSP00000293288	9606.ENSP00000381185	0	0	0	0.645	0	0.58	0.9	0.734	0.967
BCL2	MAPK1	9606.ENSP00000381185	9606.ENSP00000215832	0	0	0	0	0	0.379	0.9	0.198	0.945
BCL2	STAT3	9606.ENSP00000381185	9606.ENSP00000264657	0	0	0	0	0	0.186	0.9	0.225	0.931
BCL2	CASP8	9606.ENSP00000381185	9606.ENSP00000351273	0	0	0	0	0	0.993	0	0.43	0.996
BCL2	RXRG	9606.ENSP00000381185	9606.ENSP00000352900	0	0	0	0	0	0.064	0.9	0	0.902
BCL2	MAPK8	9606.ENSP00000381185	9606.ENSP00000378974	0	0	0	0	0	0.386	0.9	0.29	0.952
BCL2	RXRA	9606.ENSP00000381185	9606.ENSP00000419692	0	0	0	0	0	0.064	0.9	0.046	0.902
BCL2	ESR1	9606.ENSP00000381185	9606.ENSP00000405330	0	0	0	0	0.069	0.064	0.9	0.244	0.925
BCL2	NR4A1	9606.ENSP00000381185	9606.ENSP00000440864	0	0	0	0	0	0.379	0.9	0.384	0.958
BCL2	MYC	9606.ENSP00000381185	9606.ENSP00000479618	0	0	0	0	0	0.456	0.9	0.407	0.965
CASP3	MAPK8	9606.ENSP00000311032	9606.ENSP00000378974	0	0	0	0	0.055	0.294	0	0.863	0.901
CASP3	ITGB1	9606.ENSP00000311032	9606.ENSP00000379350	0	0	0	0	0.062	0	0.9	0.416	0.94
CASP3	PRKCD	9606.ENSP00000311032	9606.ENSP00000378217	0	0	0	0	0	0	0.9	0.556	0.953
CASP3	PRKDC	9606.ENSP00000311032	9606.ENSP00000313420	0	0	0	0	0	0.395	0.9	0.474	0.965
CASP3	MAPT	9606.ENSP00000311032	9606.ENSP00000340820	0	0	0	0	0	0.379	0.9	0.539	0.968
CASP3	CASP9	9606.ENSP00000311032	9606.ENSP00000330237	0	0	0	0.833	0.061	0.682	0.9	0.971	0.972
CASP3	CDKN1A	9606.ENSP00000311032	9606.ENSP00000384849	0	0	0	0	0	0.682	0.8	0.718	0.98
CASP3	PARP1	9606.ENSP00000311032	9606.ENSP00000355759	0	0	0	0	0.062	0.472	0.9	0.839	0.99
CASP3	CASP8	9606.ENSP00000311032	9606.ENSP00000351273	0	0	0	0.835	0.061	0.993	0.9	0.978	0.999

CASP8	MAPK1	9606.ENSP00000351273	9606.ENSP00000215832	0	0	0	0	0	0.482	0.9	0.778	0.987
CASP8	COL1A1	9606.ENSP00000351273	9606.ENSP00000225964	0	0	0	0	0	0	0.9	0.185	0.915
CASP8	IL1B	9606.ENSP00000351273	9606.ENSP00000263341	0	0	0	0	0.063	0.268	0.9	0.568	0.966
CASP8	COL3A1	9606.ENSP00000351273	9606.ENSP00000304408	0	0	0	0	0	0	0.9	0.216	0.918
CASP8	CASP9	9606.ENSP00000351273	9606.ENSP00000330237	0	0	0	0.707	0	0.993	0	0.956	0.994
CASP8	RELA	9606.ENSP00000351273	9606.ENSP00000384273	0	0	0	0	0.061	0	0.9	0.542	0.953
CASP8	CHUK	9606.ENSP00000351273	9606.ENSP00000359424	0	0	0	0	0	0.374	0.9	0.43	0.961
CASP8	IKBKB	9606.ENSP00000351273	9606.ENSP00000430684	0	0	0	0	0.061	0.374	0.9	0.447	0.963
CASP8	TNF	9606.ENSP00000351273	9606.ENSP00000398698	0	0	0	0	0.062	0.394	0.9	0.788	0.986
CASP9	PARP1	9606.ENSP00000330237	9606.ENSP00000355759	0	0	0	0	0.061	0.126	0.8	0.743	0.952
CAV1	EGF	9606.ENSP00000339191	9606.ENSP00000265171	0	0	0	0	0	0	0.9	0.746	0.973
CAV1	EGFR	9606.ENSP00000339191	9606.ENSP00000275493	0	0	0	0	0.335	0.393	0.9	0.844	0.992
CAV1	NOS3	9606.ENSP00000339191	9606.ENSP00000297494	0	0	0	0	0	0.379	0.9	0.915	0.994
CAV1	INSR	9606.ENSP00000339191	9606.ENSP00000303830	0	0	0	0	0	0.437	0.9	0.249	0.954
CAV1	GSK3B	9606.ENSP00000339191	9606.ENSP00000324806	0	0	0	0	0	0.057	0.9	0.282	0.926
CAV1	HSP90AA1	9606.ENSP00000339191	9606.ENSP00000335153	0	0	0	0	0	0.294	0.9	0.708	0.977
CAV1	ITGB1	9606.ENSP00000339191	9606.ENSP00000379350	0	0	0	0	0.132	0	0.9	0.392	0.942
CAV1	PTPN1	9606.ENSP00000339191	9606.ENSP00000360683	0	0	0	0	0	0.379	0.9	0.268	0.95
CAV1	TNF	9606.ENSP00000339191	9606.ENSP00000398698	0	0	0	0	0	0.348	0.9	0.507	0.965
CCL2	VEGFA	9606.ENSP00000225831	9606.ENSP00000478570	0	0	0	0	0	0	0	0.914	0.914
CCL2	ICAM1	9606.ENSP00000225831	9606.ENSP00000264832	0	0	0	0	0.149	0	0	0.919	0.929
CCL2	RELA	9606.ENSP00000225831	9606.ENSP00000384273	0	0	0	0	0	0.379	0.8	0.555	0.939
CCL2	VCAM1	9606.ENSP00000225831	9606.ENSP00000294728	0	0	0	0	0.206	0	0	0.927	0.94
CCL2	CXCL10	9606.ENSP00000225831	9606.ENSP00000305651	0	0	0	0	0.158	0	0	0.933	0.941
CCL2	IL1B	9606.ENSP00000225831	9606.ENSP00000263341	0	0	0	0	0.126	0	0	0.945	0.95
CCL2	FOS	9606.ENSP00000225831	9606.ENSP00000306245	0	0	0	0	0	0	0.9	0.719	0.97

CCL2	TNF	9606.ENSP00000225831	9606.ENSP00000398698	0	0	0	0	0.062	0	0	0.969	0.971
CCL2	JUN	9606.ENSP00000225831	9606.ENSP00000360266	0	0	0	0	0.062	0	0.9	0.792	0.978
CCL2	CXCL8	9606.ENSP00000225831	9606.ENSP00000306512	0	0	0	0	0.278	0.667	0	0.925	0.98
CCL2	STAT3	9606.ENSP00000225831	9606.ENSP00000264657	0	0	0	0	0	0	0.9	0.824	0.981
CCL2	IL6	9606.ENSP00000225831	9606.ENSP00000385675	0	0	0	0	0.267	0	0	0.977	0.982
CCL2	IL10	9606.ENSP00000225831	9606.ENSP00000412237	0	0	0	0	0	0	0.9	0.955	0.995
CCNC	FABP4	9606.ENSP00000428982	9606.ENSP00000256104	0	0	0	0	0	0	0.9	0.064	0.902
CCNC	PPARG	9606.ENSP00000428982	9606.ENSP00000287820	0	0	0	0	0	0.085	0.9	0.056	0.906
CCNC	SLC2A4	9606.ENSP00000428982	9606.ENSP00000320935	0	0	0	0	0	0	0.9	0	0.900
CCNC	CDK8	9606.ENSP00000428982	9606.ENSP00000370938	0	0	0	0	0.168	0.952	0.9	0.985	0.999
CCNC	RXRA	9606.ENSP00000428982	9606.ENSP00000419692	0	0	0	0	0.061	0.13	0.9	0	0.911
CCND1	IGF2	9606.ENSP00000227507	9606.ENSP00000391826	0	0	0	0	0.092	0	0	0.901	0.907
CCND1	EGFR	9606.ENSP00000227507	9606.ENSP00000275493	0	0	0	0	0.2	0.157	0	0.886	0.917
CCND1	MYC	9606.ENSP00000227507	9606.ENSP00000479618	0	0	0	0	0.069	0	0	0.963	0.964
CCND1	FOS	9606.ENSP00000227507	9606.ENSP00000306245	0	0	0	0	0	0	0.9	0.685	0.967
CCND1	RELA	9606.ENSP00000227507	9606.ENSP00000384273	0	0	0	0	0	0.313	0.9	0.559	0.967
CCND1	E2F1	9606.ENSP00000227507	9606.ENSP00000345571	0	0	0	0	0.064	0.132	0.9	0.713	0.973
CCND1	JUN	9606.ENSP00000227507	9606.ENSP00000360266	0	0	0	0	0	0	0.9	0.862	0.985
CCND1	GSK3B	9606.ENSP00000227507	9606.ENSP00000324806	0	0	0	0	0	0.526	0.9	0.822	0.990
CCND1	ESR1	9606.ENSP00000227507	9606.ENSP00000405330	0	0	0	0	0	0.384	0.9	0.858	0.990
CCND1	STAT3	9606.ENSP00000227507	9606.ENSP00000264657	0	0	0	0	0.049	0.457	0.9	0.894	0.993
CCND1	CDKN1A	9606.ENSP00000227507	9606.ENSP00000384849	0	0	0	0	0.084	0.993	0.9	0.857	0.999
CD40LG	PIK3CA	9606.ENSP00000359663	9606.ENSP00000263967	0	0	0	0	0	0.05	0.9	0.064	0.903
CD40LG	PIK3CG	9606.ENSP00000359663	9606.ENSP00000352121	0	0	0	0	0.088	0.05	0.9	0.19	0.920
CD40LG	PIK3R1	9606.ENSP00000359663	9606.ENSP00000428056	0	0	0	0	0	0.057	0.9	0.222	0.920
CD40LG	RELA	9606.ENSP00000359663	9606.ENSP00000384273	0	0	0	0	0	0	0.9	0.477	0.945

CD40LG	MAPK8	9606.ENSP00000359663	9606.ENSP00000378974	0	0	0	0	0	0.076	0.9	0.473	0.947
CD40LG	TNF	9606.ENSP00000359663	9606.ENSP00000398698	0	0	0	0	0.076	0	0.9	0.8	0.979
CDK8	FABP4	9606.ENSP00000370938	9606.ENSP00000256104	0	0	0	0	0	0	0.9	0	0.900
CDK8	PPARG	9606.ENSP00000370938	9606.ENSP00000287820	0	0	0	0	0	0.077	0.9	0.239	0.923
CDK8	SLC2A4	9606.ENSP00000370938	9606.ENSP00000320935	0	0	0	0	0	0	0.9	0	0.900
CDK8	RXRA	9606.ENSP00000370938	9606.ENSP00000419692	0	0	0	0	0	0.126	0.9	0.05	0.909
CDKN1A	STAT3	9606.ENSP00000384849	9606.ENSP00000264657	0	0	0	0	0.074	0.513	0.9	0.547	0.976
CDKN1A	HSP90AA1	9606.ENSP00000384849	9606.ENSP00000335153	0	0	0	0	0	0.379	0.9	0.43	0.961
CDKN1A	E2F1	9606.ENSP00000384849	9606.ENSP00000345571	0	0	0	0	0.052	0.435	0.9	0.617	0.976
CDKN1A	JUN	9606.ENSP00000384849	9606.ENSP00000360266	0	0	0	0	0.079	0.305	0.9	0.743	0.981
CDKN1A	MAPK8	9606.ENSP00000384849	9606.ENSP00000378974	0	0	0	0	0	0.379	0.9	0.593	0.972
CDKN1A	HIF1A	9606.ENSP00000384849	9606.ENSP00000437955	0	0	0	0	0	0	0.9	0.399	0.937
CDKN1A	MYC	9606.ENSP00000384849	9606.ENSP00000479618	0	0	0	0	0	0.378	0.9	0.945	0.996
CES1	MPO	9606.ENSP00000353720	9606.ENSP00000225275	0	0	0	0	0	0.07	0.9	0.295	0.928
CES1	CYP3A4	9606.ENSP00000353720	9606.ENSP00000337915	0	0.003	0	0	0.061	0	0.9	0.593	0.958
CES2	CYP3A4	9606.ENSP00000317842	9606.ENSP00000337915	0	0.002	0	0	0.054	0	0.9	0.58	0.956
CFTR	IGF2R	9606.ENSP00000003084	9606.ENSP00000349437	0	0	0	0	0	0	0.9	0.218	0.918
CFTR	F2	9606.ENSP00000003084	9606.ENSP00000308541	0	0	0	0	0	0	0.9	0.246	0.921
CFTR	EGF	9606.ENSP00000003084	9606.ENSP00000265171	0	0	0	0	0	0	0.9	0.384	0.935
CFTR	EGFR	9606.ENSP00000003084	9606.ENSP00000275493	0	0	0	0	0	0.051	0.9	0.383	0.936
CFTR	HSPA8	9606.ENSP00000003084	9606.ENSP00000432083	0	0	0	0	0.062	0.404	0.9	0.562	0.972
CHRM3	F2	9606.ENSP00000255380	9606.ENSP00000308541	0	0	0	0	0	0	0.9	0.041	0.900
CHRM3	PIK3CA	9606.ENSP00000255380	9606.ENSP00000263967	0	0	0	0	0	0	0.9	0.049	0.900
CHRM3	PIK3R1	9606.ENSP00000255380	9606.ENSP00000428056	0	0	0	0	0	0	0.9	0	0.900
CHRM3	GCGR	9606.ENSP00000255380	9606.ENSP00000383558	0	0	0	0	0	0	0.9	0.096	0.905
CHRM3	HTR2A	9606.ENSP00000255380	9606.ENSP00000437737	0	0	0	0.627	0.081	0	0.9	0.318	0.914

CHRM3	F2RL1	9606.ENSP00000255380	9606.ENSP00000296677	0	0	0	0	0	0	0.9	0.199	0.916
CHUK	IL1A	9606.ENSP00000359424	9606.ENSP00000263339	0	0	0	0	0.061	0	0.9	0.22	0.920
CHUK	PRKCB	9606.ENSP00000359424	9606.ENSP00000305355	0	0	0	0.565	0	0.717	0.9	0.254	0.973
CHUK	PRKCE	9606.ENSP00000359424	9606.ENSP00000306124	0	0	0	0.557	0.062	0.071	0.9	0.231	0.913
CHUK	MTOR	9606.ENSP00000359424	9606.ENSP00000354558	0	0	0	0	0.058	0.882	0	0.197	0.903
CHUK	PRKCA	9606.ENSP00000359424	9606.ENSP00000408695	0	0	0	0.568	0	0.358	0.9	0.31	0.941
CHUK	TNF	9606.ENSP00000359424	9606.ENSP00000398698	0	0	0	0	0.061	0.374	0.9	0.592	0.972
CHUK	IKBKB	9606.ENSP00000359424	9606.ENSP00000430684	0	0	0	0.942	0	0.91	0.9	0.953	0.991
CHUK	RELA	9606.ENSP00000359424	9606.ENSP00000384273	0	0	0	0	0.067	0.871	0.9	0.705	0.995
CNR2	DRD4	9606.ENSP00000363596	9606.ENSP00000176183	0	0	0	0	0	0	0.9	0.513	0.949
CNR2	CXCL10	9606.ENSP00000363596	9606.ENSP00000305651	0	0	0	0	0	0	0.9	0.14	0.910
CNR2	CXCL8	9606.ENSP00000363596	9606.ENSP00000306512	0	0	0	0	0	0	0.9	0.25	0.921
CNR2	CXCR2	9606.ENSP00000363596	9606.ENSP00000319635	0	0	0	0	0.087	0	0.9	0.094	0.910
CNR2	DRD2	9606.ENSP00000363596	9606.ENSP00000354859	0	0	0	0	0	0	0.9	0.534	0.951
CNR2	HTR1B	9606.ENSP00000363596	9606.ENSP00000358963	0	0	0	0.582	0	0	0.9	0.167	0.905
CNR2	OPRM1	9606.ENSP00000363596	9606.ENSP00000394624	0	0	0	0.567	0.063	0	0.9	0.669	0.929
COL1A1	MMP2	9606.ENSP00000225964	9606.ENSP00000219070	0	0	0	0	0.89	0.379	0	0.6	0.970
COL1A1	MSR1	9606.ENSP00000225964	9606.ENSP00000262101	0	0	0	0.595	0	0	0.9	0.187	0.906
COL1A1	ITGB1	9606.ENSP00000225964	9606.ENSP00000379350	0	0	0	0	0.096	0.379	0.9	0.52	0.969
COL1A1	COL3A1	9606.ENSP00000225964	9606.ENSP00000304408	0	0	0.439	0.951	0.944	0	0.9	0.901	0.994
COL3A1	MSR1	9606.ENSP00000304408	9606.ENSP00000262101	0	0	0	0.587	0.097	0	0.9	0.079	0.907
COL3A1	ITGB1	9606.ENSP00000304408	9606.ENSP00000379350	0	0	0	0	0.09	0	0.9	0.405	0.941
CPB2	PLG	9606.ENSP00000181383	9606.ENSP00000308938	0	0	0	0	0.326	0.432	0	0.799	0.916
CPB2	F2	9606.ENSP00000181383	9606.ENSP00000308541	0	0	0	0	0.605	0.432	0.9	0.7	0.992
CPT1A	RELA	9606.ENSP00000265641	9606.ENSP00000384273	0	0	0	0	0	0	0.9	0.041	0.900
CPT1A	JUN	9606.ENSP00000265641	9606.ENSP00000360266	0	0	0	0	0	0	0.9	0.145	0.91

CPT1A	RXRA	9606.ENSP00000265641	9606.ENSP00000419692	0	0	0	0	0.062	0.05	0.9	0.345	0.933
CPT1A	FABP1	9606.ENSP00000265641	9606.ENSP00000295834	0	0	0	0	0.061	0	0.9	0.592	0.958
CPT1A	PPARA	9606.ENSP00000265641	9606.ENSP00000385523	0	0	0	0	0.062	0.05	0.9	0.751	0.974
CRP	CXCL8	9606.ENSP00000255030	9606.ENSP00000306512	0	0	0	0	0	0	0	0.909	0.909
CRP	IL6	9606.ENSP00000255030	9606.ENSP00000385675	0	0	0	0	0	0	0	0.97	0.970
CTSB	CTSD	9606.ENSP00000345672	9606.ENSP00000236671	0	0	0	0	0.228	0.45	0	0.936	0.970
CTSD	TPO	9606.ENSP00000236671	9606.ENSP00000318820	0	0	0	0	0.088	0	0.9	0.046	0.905
CTSD	HPSE	9606.ENSP00000236671	9606.ENSP00000384262	0	0	0	0	0	0	0.9	0.112	0.907
CTSD	SLPI	9606.ENSP00000236671	9606.ENSP00000342082	0	0	0	0	0.072	0	0.9	0.158	0.915
CTSD	MMP9	9606.ENSP00000236671	9606.ENSP00000361405	0	0	0	0	0.08	0	0.9	0.445	0.944
CTSD	ESR1	9606.ENSP00000236671	9606.ENSP00000405330	0	0	0	0	0.087	0	0.9	0.587	0.959
CTSD	CTSS	9606.ENSP00000236671	9606.ENSP00000357981	0	0	0	0	0.063	0.08	0.9	0.589	0.959
CTSS	MMP9	9606.ENSP00000357981	9606.ENSP00000361405	0	0	0	0	0.134	0	0.9	0.319	0.935
CXCL10	DRD4	9606.ENSP00000305651	9606.ENSP00000176183	0	0	0	0	0	0	0.9	0	0.900
CXCL10	IFNG	9606.ENSP00000305651	9606.ENSP00000229135	0	0	0	0	0.118	0	0	0.893	0.902
CXCL10	STAT3	9606.ENSP00000305651	9606.ENSP00000264657	0	0	0	0	0	0	0.9	0.736	0.972
CXCL10	HTR1B	9606.ENSP00000305651	9606.ENSP00000358963	0	0	0	0	0	0	0.9	0	0.900
CXCL10	DRD2	9606.ENSP00000305651	9606.ENSP00000354859	0	0	0	0	0	0	0.9	0.064	0.902
CXCL10	OPRM1	9606.ENSP00000305651	9606.ENSP00000394624	0	0	0	0	0	0	0.9	0.14	0.910
CXCL10	STAT1	9606.ENSP00000305651	9606.ENSP00000354394	0	0	0	0	0.707	0	0	0.84	0.951
CXCL10	CXCR2	9606.ENSP00000305651	9606.ENSP00000319635	0	0	0	0	0	0	0.9	0.672	0.965
CXCL10	CXCL8	9606.ENSP00000305651	9606.ENSP00000306512	0	0	0	0	0.144	0	0.9	0.869	0.987
CXCL10	IL10	9606.ENSP00000305651	9606.ENSP00000412237	0	0	0	0	0.083	0	0.9	0.913	0.991
CXCL8	DRD4	9606.ENSP00000306512	9606.ENSP00000176183	0	0	0	0	0	0	0.9	0.136	0.909
CXCL8	IL1B	9606.ENSP00000306512	9606.ENSP00000263341	0	0	0	0	0.676	0	0	0.977	0.992
CXCL8	STAT3	9606.ENSP00000306512	9606.ENSP00000264657	0	0	0	0	0	0	0.9	0.838	0.983

CXCL8	ICAM1	9606.ENSP00000306512	9606.ENSP00000264832	0	0	0	0	0.214	0	0	0.912	0.928
CXCL8	HTR1B	9606.ENSP00000306512	9606.ENSP00000358963	0	0	0	0	0	0	0.9	0.07	0.903
CXCL8	DRD2	9606.ENSP00000306512	9606.ENSP00000354859	0	0	0	0	0	0	0.9	0.167	0.913
CXCL8	OPRM1	9606.ENSP00000306512	9606.ENSP00000394624	0	0	0	0	0	0	0.9	0.293	0.926
CXCL8	MMP9	9606.ENSP00000306512	9606.ENSP00000361405	0	0	0	0	0.134	0.379	0	0.885	0.933
CXCL8	VEGFA	9606.ENSP00000306512	9606.ENSP00000478570	0	0	0	0	0.065	0	0	0.953	0.954
CXCL8	PTGS2	9606.ENSP00000306512	9606.ENSP00000356438	0	0	0	0	0.753	0	0	0.839	0.958
CXCL8	JUN	9606.ENSP00000306512	9606.ENSP00000360266	0	0	0	0	0.095	0.294	0.8	0.799	0.971
CXCL8	TNF	9606.ENSP00000306512	9606.ENSP00000398698	0	0	0	0	0.156	0	0	0.97	0.973
CXCL8	IL6	9606.ENSP00000306512	9606.ENSP00000385675	0	0	0	0	0.581	0	0	0.966	0.985
CXCL8	RELA	9606.ENSP00000306512	9606.ENSP00000384273	0	0	0	0	0.065	0.342	0.9	0.817	0.987
CXCL8	IL10	9606.ENSP00000306512	9606.ENSP00000412237	0	0	0	0	0.061	0	0.9	0.962	0.996
CXCL8	CXCR2	9606.ENSP00000306512	9606.ENSP00000319635	0	0	0	0	0.064	0.667	0.9	0.937	0.997
CXCR2	DRD4	9606.ENSP00000319635	9606.ENSP00000176183	0	0	0	0.576	0	0	0.9	0.053	0.900
CXCR2	HTR1B	9606.ENSP00000319635	9606.ENSP00000358963	0	0	0	0.568	0	0	0.9	0.064	0.901
CXCR2	DRD2	9606.ENSP00000319635	9606.ENSP00000354859	0	0	0	0.57	0	0	0.9	0.073	0.901
CXCR2	OPRM1	9606.ENSP00000319635	9606.ENSP00000394624	0	0	0	0.739	0	0	0.9	0.355	0.908
CYP19A1	SULT1E1	9606.ENSP00000379683	9606.ENSP00000226444	0	0	0	0	0	0	0.9	0.64	0.962
CYP19A1	CYP3A4	9606.ENSP00000379683	9606.ENSP00000337915	0	0	0.419	0.577	0	0	0.9	0.609	0.937
CYP19A1	CYP1A1	9606.ENSP00000379683	9606.ENSP00000369050	0	0	0.409	0.563	0	0	0.9	0.68	0.941
CYP19A1	ESR1	9606.ENSP00000379683	9606.ENSP00000405330	0	0	0	0	0	0.085	0	0.96	0.962
CYP1A1	SULT1E1	9606.ENSP00000369050	9606.ENSP00000226444	0	0	0	0	0.063	0	0.9	0.445	0.943
CYP1A1	FABP1	9606.ENSP00000369050	9606.ENSP00000295834	0	0	0	0	0.129	0	0.9	0.108	0.915
CYP1A1	CYP2A6	9606.ENSP00000369050	9606.ENSP00000301141	0	0	0.434	0.815	0.087	0	0.9	0.802	0.924
CYP1A1	GSTM1	9606.ENSP00000369050	9606.ENSP00000311469	0	0	0	0	0.061	0.077	0.65	0.892	0.962
CYP1A1	CYP3A4	9606.ENSP00000369050	9606.ENSP00000337915	0	0	0.443	0.605	0.129	0	0.9	0.895	0.95

CYP1A1	CYP2D6	9606.ENSP00000369050	9606.ENSP00000353820	0	0	0.41	0.809	0.062	0	0.9	0.837	0.923
CYP1A1	HSD11B1	9606.ENSP00000369050	9606.ENSP00000355995	0	0	0	0	0.145	0.131	0.9	0.17	0.930
CYP1A1	GSTP1	9606.ENSP00000369050	9606.ENSP00000381607	0	0	0	0	0	0.077	0.65	0.738	0.908
CYP1A1	RXRA	9606.ENSP00000369050	9606.ENSP00000419692	0	0	0	0	0.051	0.085	0.9	0.212	0.922
CYP1A1	PPARA	9606.ENSP00000369050	9606.ENSP00000385523	0	0	0	0	0.063	0.085	0.9	0.359	0.937
CYP1A2	CYP2A6	9606.ENSP00000342007	9606.ENSP00000301141	0	0	0.435	0.776	0.108	0	0.9	0.912	0.932
CYP1A2	CYP3A4	9606.ENSP00000342007	9606.ENSP00000337915	0	0	0.442	0.604	0.139	0	0.9	0.935	0.952
CYP1A2	XDH	9606.ENSP00000342007	9606.ENSP00000368727	0	0	0	0	0.061	0	0.9	0.096	0.907
CYP1A2	NR1I3	9606.ENSP00000342007	9606.ENSP00000356959	0	0	0	0	0.076	0.085	0.9	0.646	0.966
CYP2A6	HSD11B1	9606.ENSP00000301141	9606.ENSP00000355995	0	0	0	0	0.11	0.134	0.9	0.081	0.919
CYP2A6	CYP2D6	9606.ENSP00000301141	9606.ENSP00000353820	0	0	0.422	0.87	0.107	0	0.9	0.913	0.922
CYP2A6	CYP3A4	9606.ENSP00000301141	9606.ENSP00000337915	0	0	0.44	0.633	0.14	0	0.9	0.904	0.949
CYP2D6	CYP3A4	9606.ENSP00000353820	9606.ENSP00000337915	0	0	0.421	0.617	0.089	0	0.9	0.935	0.948
CYP2D6	MAOA	9606.ENSP00000353820	9606.ENSP00000340684	0	0	0	0	0.063	0	0.9	0.522	0.951
CYP2D6	HSD11B1	9606.ENSP00000353820	9606.ENSP00000355995	0	0	0	0	0.062	0.193	0.9	0.163	0.928
CYP2D6	MAOB	9606.ENSP00000353820	9606.ENSP00000367309	0	0	0	0	0.063	0	0.9	0.479	0.946
CYP3A4	SULT1E1	9606.ENSP00000337915	9606.ENSP00000226444	0	0	0	0	0.086	0	0.9	0.429	0.943
CYP3A4	NR1I2	9606.ENSP00000337915	9606.ENSP00000336528	0	0	0	0	0.134	0.085	0	0.955	0.962
CYP3A4	MAOB	9606.ENSP00000337915	9606.ENSP00000367309	0	0	0	0	0.063	0	0.9	0.267	0.925
CYP3A4	HSD11B1	9606.ENSP00000337915	9606.ENSP00000355995	0	0	0	0	0.113	0.059	0.9	0.255	0.929
CYP3A4	MAOA	9606.ENSP00000337915	9606.ENSP00000340684	0	0	0	0	0.063	0	0.9	0.34	0.932
DGAT1	OLR1	9606.ENSP00000482264	9606.ENSP00000309124	0	0	0	0	0	0	0.9	0.156	0.912
DRD2	DRD4	9606.ENSP00000354859	9606.ENSP00000176183	0	0	0	0.855	0	0.379	0.9	0.885	0.943
DRD2	SLC6A3	9606.ENSP00000354859	9606.ENSP00000270349	0	0	0	0	0.062	0.454	0.8	0.917	0.99
DRD2	HTR1B	9606.ENSP00000354859	9606.ENSP00000358963	0	0	0	0.79	0.062	0	0.9	0.657	0.915
DRD2	OPRM1	9606.ENSP00000354859	9606.ENSP00000394624	0	0	0	0.583	0	0	0.9	0.789	0.932

DRD4	HTR1B	9606.ENSP00000176183	9606.ENSP00000358963	0	0	0	0.746	0	0	0.9	0.673	0.916
DRD4	OPRM1	9606.ENSP00000176183	9606.ENSP00000394624	0	0	0	0.587	0	0	0.9	0.735	0.929
E2F1	SORT1	9606.ENSP00000345571	9606.ENSP00000256637	0	0	0	0	0	0	0.9	0	0.900
EGF	SERPINE1	9606.ENSP00000265171	9606.ENSP00000223095	0	0	0	0	0	0	0.9	0.798	0.978
EGF	PIK3CA	9606.ENSP00000265171	9606.ENSP00000263967	0	0	0	0	0	0	0.9	0.583	0.956
EGF	STAT3	9606.ENSP00000265171	9606.ENSP00000264657	0	0	0	0	0	0	0.9	0.853	0.984
EGF	PRKCB	9606.ENSP00000265171	9606.ENSP00000305355	0	0	0	0	0	0	0.9	0.266	0.923
EGF	HSPA8	9606.ENSP00000265171	9606.ENSP00000432083	0	0	0	0	0	0	0.9	0.309	0.927
EGF	PIK3R1	9606.ENSP00000265171	9606.ENSP00000428056	0	0	0	0	0	0	0.9	0.389	0.936
EGF	IGF2R	9606.ENSP00000265171	9606.ENSP00000349437	0	0	0	0	0	0	0.9	0.421	0.939
EGF	PRKCA	9606.ENSP00000265171	9606.ENSP00000408695	0	0	0	0	0	0	0.9	0.473	0.945
EGF	PTPN1	9606.ENSP00000265171	9606.ENSP00000360683	0	0	0	0	0	0	0.9	0.532	0.951
EGF	STAT1	9606.ENSP00000265171	9606.ENSP00000354394	0	0	0	0	0	0	0.9	0.558	0.953
EGF	HSP90AA1	9606.ENSP00000265171	9606.ENSP00000335153	0	0	0	0	0	0.064	0.9	0.557	0.954
EGF	ITGB1	9606.ENSP00000265171	9606.ENSP00000379350	0	0	0	0	0	0.085	0.9	0.569	0.957
EGF	PLG	9606.ENSP00000265171	9606.ENSP00000308938	0	0	0	0	0	0	0.9	0.661	0.964
EGF	IGF2	9606.ENSP00000265171	9606.ENSP00000391826	0	0	0	0	0	0	0.9	0.672	0.965
EGF	MTOR	9606.ENSP00000265171	9606.ENSP00000354558	0	0	0	0	0	0	0.9	0.668	0.965
EGF	VEGFA	9606.ENSP00000265171	9606.ENSP00000478570	0	0	0	0	0	0	0.9	0.937	0.993
EGF	EGFR	9606.ENSP00000265171	9606.ENSP00000275493	0	0	0	0	0.16	0.933	0.9	0.972	0.999
EGFR	MAPK1	9606.ENSP00000275493	9606.ENSP00000215832	0	0	0	0.577	0	0.472	0.9	0.887	0.965
EGFR	PIK3CA	9606.ENSP00000275493	9606.ENSP00000263967	0	0	0	0	0	0.37	0.9	0.866	0.99
EGFR	STAT3	9606.ENSP00000275493	9606.ENSP00000264657	0	0	0	0	0	0.753	0.9	0.927	0.998
EGFR	FOS	9606.ENSP00000275493	9606.ENSP00000306245	0	0	0	0	0	0.533	0	0.794	0.900
EGFR	IL6	9606.ENSP00000275493	9606.ENSP00000385675	0	0	0	0	0	0	0.6	0.811	0.921
EGFR	IGF2R	9606.ENSP00000275493	9606.ENSP00000349437	0	0	0	0	0.062	0	0.9	0.441	0.942

EGFR	ESR1	9606.ENSP00000275493	9606.ENSP00000405330	0	0	0	0	0	0.402	0	0.908	0.943
EGFR	PRKCB	9606.ENSP00000275493	9606.ENSP00000305355	0	0	0	0.553	0.049	0.403	0.9	0.304	0.945
EGFR	PRKCA	9606.ENSP00000275493	9606.ENSP00000408695	0	0	0	0.557	0.049	0.44	0.9	0.676	0.959
EGFR	HSPA8	9606.ENSP00000275493	9606.ENSP00000432083	0	0	0	0	0	0.393	0.9	0.386	0.959
EGFR	ITGB1	9606.ENSP00000275493	9606.ENSP00000379350	0	0	0	0	0.097	0.416	0.9	0.454	0.967
EGFR	HIF1A	9606.ENSP00000275493	9606.ENSP00000437955	0	0	0	0	0	0	0.9	0.743	0.973
EGFR	VEGFA	9606.ENSP00000275493	9606.ENSP00000478570	0	0	0	0	0.089	0	0.6	0.947	0.979
EGFR	STAT1	9606.ENSP00000275493	9606.ENSP00000354394	0	0	0	0	0.061	0.42	0.9	0.764	0.985
EGFR	MTOR	9606.ENSP00000275493	9606.ENSP00000354558	0	0	0	0	0.061	0.204	0.9	0.843	0.986
EGFR	PTPN1	9606.ENSP00000275493	9606.ENSP00000360683	0	0	0	0	0	0.47	0.9	0.798	0.988
EGFR	HSP90AA1	9606.ENSP00000275493	9606.ENSP00000335153	0	0	0	0	0	0.487	0.9	0.817	0.989
EGFR	PIK3R1	9606.ENSP00000275493	9606.ENSP00000428056	0	0	0	0	0	0.882	0.9	0.581	0.994
ERN1	HSPA5	9606.ENSP00000401445	9606.ENSP00000324173	0	0	0	0	0	0.521	0.9	0.948	0.997
ESR1	MAPK1	9606.ENSP00000405330	9606.ENSP00000215832	0	0	0	0	0	0.399	0.9	0.57	0.971
ESR1	NR3C1	9606.ENSP00000405330	9606.ENSP00000231509	0	0	0	0.66	0	0	0.9	0.751	0.925
ESR1	HSPB1	9606.ENSP00000405330	9606.ENSP00000248553	0	0	0	0	0	0.336	0.9	0.414	0.957
ESR1	PIK3CA	9606.ENSP00000405330	9606.ENSP00000263967	0	0	0	0	0	0.379	0.9	0.669	0.977
ESR1	IGF1R	9606.ENSP00000405330	9606.ENSP00000268035	0	0	0	0	0	0.407	0.9	0.835	0.989
ESR1	NOS3	9606.ENSP00000405330	9606.ENSP00000297494	0	0	0	0	0	0.472	0.9	0.749	0.985
ESR1	FOS	9606.ENSP00000405330	9606.ENSP00000306245	0	0	0	0	0	0.475	0.9	0.688	0.982
ESR1	TERT	9606.ENSP00000405330	9606.ENSP00000309572	0	0	0	0	0	0.043	0.9	0.695	0.968
ESR1	HSP90AA1	9606.ENSP00000405330	9606.ENSP00000335153	0	0	0	0	0	0.474	0.9	0.633	0.979
ESR1	STAT1	9606.ENSP00000405330	9606.ENSP00000354394	0	0	0	0	0	0.448	0.9	0.458	0.967
ESR1	JUN	9606.ENSP00000405330	9606.ENSP00000360266	0	0	0	0	0	0.472	0.9	0.646	0.979
ESR1	MAPK8	9606.ENSP00000405330	9606.ENSP00000378974	0	0	0	0	0	0	0.8	0.559	0.908
ESR1	ESRRA	9606.ENSP00000405330	9606.ENSP00000384851	0	0	0	0.849	0	0.379	0.9	0.668	0.941

ESR1	PIK3R1	9606.ENSP00000405330	9606.ENSP00000428056	0	0	0	0	0.063	0.404	0.9	0.418	0.963
ESR1	MYC	9606.ENSP00000405330	9606.ENSP00000479618	0	0	0	0	0	0.442	0.9	0.721	0.983
ESRRA	NR3C1	9606.ENSP00000384851	9606.ENSP00000231509	0	0	0	0.746	0	0	0.9	0.348	0.908
ESRRA	STAT1	9606.ENSP00000384851	9606.ENSP00000354394	0	0	0	0	0	0.053	0.9	0.109	0.908
ESRRA	PPARA	9606.ENSP00000384851	9606.ENSP00000385523	0	0	0	0.616	0.05	0	0.9	0.719	0.927
F2	MAPK1	9606.ENSP00000308541	9606.ENSP00000215832	0	0	0	0	0	0.053	0.9	0.331	0.931
F2	PIK3CA	9606.ENSP00000308541	9606.ENSP00000263967	0	0	0	0	0	0	0.9	0.074	0.903
F2	F2RL1	9606.ENSP00000308541	9606.ENSP00000296677	0	0	0	0	0.061	0.348	0.9	0.413	0.959
F2	PIK3R1	9606.ENSP00000308541	9606.ENSP00000428056	0	0	0	0	0	0	0.9	0.085	0.904
F2	PPARA	9606.ENSP00000308541	9606.ENSP00000385523	0	0	0	0	0.063	0	0.9	0.106	0.908
F2	HTR2A	9606.ENSP00000308541	9606.ENSP00000437737	0	0	0	0	0	0	0.9	0.125	0.908
F2	GCGR	9606.ENSP00000308541	9606.ENSP00000383558	0	0	0	0	0.12	0	0.9	0.092	0.913
F2	IGF2	9606.ENSP00000308541	9606.ENSP00000391826	0	0	0	0	0.169	0	0.9	0.149	0.923
F2	IGFBP3	9606.ENSP00000308541	9606.ENSP00000370473	0	0	0	0	0.1	0.379	0.9	0.124	0.944
F2	F3	9606.ENSP00000308541	9606.ENSP00000334145	0	0	0	0	0	0	0.9	0.933	0.993
F2RL1	PIK3CA	9606.ENSP00000296677	9606.ENSP00000263967	0	0	0	0	0	0	0.9	0.044	0.900
F2RL1	HTR2A	9606.ENSP00000296677	9606.ENSP00000437737	0	0	0	0	0	0	0.9	0.05	0.900
F2RL1	GCGR	9606.ENSP00000296677	9606.ENSP00000383558	0	0	0	0	0	0	0.9	0.062	0.902
F2RL1	PIK3R1	9606.ENSP00000296677	9606.ENSP00000428056	0	0	0	0	0	0	0.9	0.151	0.911
F3	PLG	9606.ENSP00000334145	9606.ENSP00000308938	0	0	0	0	0	0.379	0	0.866	0.913
FABP1	FABP4	9606.ENSP00000295834	9606.ENSP00000256104	0	0	0	0	0.062	0	0.9	0.698	0.969
FABP1	RELA	9606.ENSP00000295834	9606.ENSP00000384273	0	0	0	0	0	0	0.9	0.047	0.900
FABP1	JUN	9606.ENSP00000295834	9606.ENSP00000360266	0	0	0	0	0	0	0.9	0.121	0.908
FABP1	FDFT1	9606.ENSP00000295834	9606.ENSP00000480828	0	0	0	0	0	0	0.9	0.145	0.910
FABP1	RXRA	9606.ENSP00000295834	9606.ENSP00000419692	0	0	0	0	0.061	0.064	0.9	0.265	0.926
FABP1	PPARA	9606.ENSP00000295834	9606.ENSP00000385523	0	0	0	0	0.064	0.472	0.9	0.788	0.988

FABP4	RXRA	9606.ENSP00000256104	9606.ENSP00000419692	0	0	0	0	0.061	0.064	0.9	0.455	0.945
FABP4	PPARG	9606.ENSP00000256104	9606.ENSP00000287820	0	0	0	0	0.087	0.064	0.9	0.915	0.991
FDFT1	SQLE	9606.ENSP00000480828	9606.ENSP00000265896	0.135	0	0	0	0.569	0	0.9	0.945	0.997
FDFT1	PPARA	9606.ENSP00000480828	9606.ENSP00000385523	0	0	0	0	0	0	0.9	0.267	0.923
FDFT1	RXRA	9606.ENSP00000480828	9606.ENSP00000419692	0	0	0	0	0	0	0.9	0.107	0.906
FGF1	MAPK1	9606.ENSP00000480791	9606.ENSP00000215832	0	0	0	0	0	0	0.9	0.397	0.937
FGF1	PIK3CA	9606.ENSP00000480791	9606.ENSP00000263967	0	0	0	0	0	0	0.9	0.129	0.909
FGF1	PIK3R1	9606.ENSP00000480791	9606.ENSP00000428056	0	0	0	0	0.062	0	0.9	0.228	0.921
FLT1	PIK3CA	9606.ENSP00000282397	9606.ENSP00000263967	0	0	0	0	0	0.263	0.9	0.427	0.954
FLT1	PIK3R1	9606.ENSP00000282397	9606.ENSP00000428056	0	0	0	0	0	0.719	0.9	0.187	0.975
FLT1	VEGFA	9606.ENSP00000282397	9606.ENSP00000478570	0	0	0	0	0.061	0.556	0.9	0.984	0.999
FOS	MAPK1	9606.ENSP00000306245	9606.ENSP00000215832	0	0	0	0	0	0.523	0.9	0.852	0.992
FOS	HMOX1	9606.ENSP00000306245	9606.ENSP00000216117	0	0	0	0	0	0	0.9	0.482	0.946
FOS	NR3C1	9606.ENSP00000306245	9606.ENSP00000231509	0	0	0	0	0.055	0.299	0.9	0.634	0.972
FOS	IL1A	9606.ENSP00000306245	9606.ENSP00000263339	0	0	0	0	0.062	0	0.9	0.425	0.941
FOS	STAT3	9606.ENSP00000306245	9606.ENSP00000264657	0	0	0	0	0	0.423	0.9	0.811	0.988
FOS	PPARG	9606.ENSP00000306245	9606.ENSP00000287820	0	0	0	0	0	0.098	0.9	0.683	0.968
FOS	MYC	9606.ENSP00000306245	9606.ENSP00000479618	0	0	0	0	0.064	0.442	0	0.851	0.915
FOS	NFE2L2	9606.ENSP00000306245	9606.ENSP00000380252	0	0	0	0	0	0.051	0.9	0.426	0.94
FOS	RELA	9606.ENSP00000306245	9606.ENSP00000384273	0	0	0	0	0	0.379	0.9	0.539	0.968
FOS	TNF	9606.ENSP00000306245	9606.ENSP00000398698	0	0	0	0	0	0	0.9	0.819	0.981
FOS	IL6	9606.ENSP00000306245	9606.ENSP00000385675	0	0	0	0	0.088	0	0.9	0.819	0.982
FOS	MAPK8	9606.ENSP00000306245	9606.ENSP00000378974	0	0	0	0	0	0.26	0.9	0.885	0.99
FOS	JUN	9606.ENSP00000306245	9606.ENSP00000360266	0	0	0	0	0.656	0.879	0.9	0.975	0.999
GCGR	PIK3CA	9606.ENSP00000383558	9606.ENSP00000263967	0	0	0	0	0	0	0.9	0.042	0.900
GCGR	HTR7	9606.ENSP00000383558	9606.ENSP00000337949	0	0	0	0	0	0	0.9	0.101	0.906

GCGR	HTR2A	9606.ENSP00000383558	9606.ENSP00000437737	0	0	0	0	0	0	0.9	0.05	0.900
GCGR	PIK3R1	9606.ENSP00000383558	9606.ENSP00000428056	0	0	0	0	0	0	0.9	0	0.900
GLB1	MAPK1	9606.ENSP00000306920	9606.ENSP00000215832	0	0	0	0	0	0	0.9	0.086	0.904
GLB1	MPO	9606.ENSP00000306920	9606.ENSP00000225275	0	0	0	0	0	0	0.9	0.057	0.901
GLB1	TTR	9606.ENSP00000306920	9606.ENSP00000237014	0	0	0	0	0	0	0.9	0.058	0.901
GLB1	PRKCD	9606.ENSP00000306920	9606.ENSP00000378217	0	0	0	0	0	0	0.9	0.05	0.900
GSK3B	NR3C1	9606.ENSP00000324806	9606.ENSP00000231509	0	0	0	0	0	0.085	0.9	0.432	0.943
GSK3B	PIN1	9606.ENSP00000324806	9606.ENSP00000247970	0	0	0	0	0.061	0.085	0.9	0.115	0.913
GSK3B	TP63	9606.ENSP00000324806	9606.ENSP00000264731	0	0	0	0	0	0	0.9	0.101	0.906
GSK3B	STAT1	9606.ENSP00000324806	9606.ENSP00000354394	0	0	0	0	0.062	0.124	0.9	0.209	0.926
GSK3B	JUN	9606.ENSP00000324806	9606.ENSP00000360266	0	0	0	0	0	0.541	0.9	0.574	0.978
GSK3B	MYC	9606.ENSP00000324806	9606.ENSP00000479618	0	0	0	0	0.063	0.391	0.9	0.732	0.982
GSK3B	MAPT	9606.ENSP00000324806	9606.ENSP00000340820	0	0	0	0	0.061	0.379	0.9	0.804	0.987
GSTM1	GSTM2	9606.ENSP00000311469	9606.ENSP00000241337	0	0	0.447	0.981	0.83	0.379	0.65	0.831	0.960
HIF1A	MAPK1	9606.ENSP00000437955	9606.ENSP00000215832	0	0	0	0	0	0.294	0.8	0.455	0.916
HIF1A	NR3C1	9606.ENSP00000437955	9606.ENSP00000231509	0	0	0	0	0	0.095	0.9	0.326	0.933
HIF1A	STAT3	9606.ENSP00000437955	9606.ENSP00000264657	0	0	0	0	0.061	0.434	0.9	0.781	0.986
HIF1A	NOS3	9606.ENSP00000437955	9606.ENSP00000297494	0	0	0	0	0	0	0.9	0.706	0.969
HIF1A	HSP90AA1	9606.ENSP00000437955	9606.ENSP00000335153	0	0	0	0	0	0.472	0.9	0.597	0.976
HIF1A	JUN	9606.ENSP00000437955	9606.ENSP00000360266	0	0	0	0	0	0.379	0.9	0.561	0.970
HIF1A	VEGFA	9606.ENSP00000437955	9606.ENSP00000478570	0	0	0	0	0.062	0.348	0.9	0.947	0.996
HMOX1	NQO1	9606.ENSP00000216117	9606.ENSP00000319788	0	0	0	0	0.056	0	0	0.951	0.952
HMOX1	JUN	9606.ENSP00000216117	9606.ENSP00000360266	0	0	0	0	0	0	0.9	0.784	0.977
HMOX1	NFE2L2	9606.ENSP00000216117	9606.ENSP00000380252	0	0	0	0	0	0	0.9	0.968	0.996
HPSE	SLPI	9606.ENSP00000384262	9606.ENSP00000342082	0	0	0	0	0.061	0	0.9	0	0.902
HSD11B1	PPARG	9606.ENSP00000355995	9606.ENSP00000287820	0	0	0	0	0.061	0	0.9	0.462	0.945

HSD11B1	RXRA	9606.ENSP00000355995	9606.ENSP00000419692	0	0	0	0	0	0	0.9	0.094	0.905
HSP90AA1	MAPK1	9606.ENSP00000335153	9606.ENSP00000215832	0	0	0	0	0.06	0.524	0.9	0.605	0.979
HSP90AA1	MMP2	9606.ENSP00000335153	9606.ENSP00000219070	0	0	0	0	0	0.379	0	0.878	0.921
HSP90AA1	IL2	9606.ENSP00000335153	9606.ENSP00000226730	0	0	0	0	0	0	0.9	0.416	0.939
HSP90AA1	IFNG	9606.ENSP00000335153	9606.ENSP00000229135	0	0	0	0	0	0	0.9	0.255	0.922
HSP90AA1	NR3C1	9606.ENSP00000335153	9606.ENSP00000231509	0	0	0	0	0	0.474	0.9	0.71	0.983
HSP90AA1	PIK3CA	9606.ENSP00000335153	9606.ENSP00000263967	0	0	0	0	0	0.063	0.9	0.38	0.936
HSP90AA1	STAT3	9606.ENSP00000335153	9606.ENSP00000264657	0	0	0	0	0	0.379	0.9	0.745	0.982
HSP90AA1	NOS3	9606.ENSP00000335153	9606.ENSP00000297494	0	0	0	0	0.06	0.379	0.9	0.873	0.991
HSP90AA1	TERT	9606.ENSP00000335153	9606.ENSP00000309572	0	0	0	0	0	0.446	0.9	0.704	0.982
HSP90AA1	HSPA5	9606.ENSP00000335153	9606.ENSP00000324173	0	0	0	0	0.637	0.412	0	0.85	0.965
HSP90AA1	PIK3R1	9606.ENSP00000335153	9606.ENSP00000428056	0	0	0	0	0.061	0	0.9	0.232	0.921
HSP90AA1	PRKCA	9606.ENSP00000335153	9606.ENSP00000408695	0	0	0	0	0	0.32	0.9	0.211	0.941
HSP90AA1	PPARA	9606.ENSP00000335153	9606.ENSP00000385523	0	0	0	0	0	0.474	0.9	0.239	0.956
HSP90AA1	MTOR	9606.ENSP00000335153	9606.ENSP00000354558	0	0	0	0	0	0.063	0.9	0.615	0.96
HSP90AA1	NR3C2	9606.ENSP00000335153	9606.ENSP00000350815	0	0	0	0	0	0.474	0.9	0.425	0.967
HSP90AA1	VEGFA	9606.ENSP00000335153	9606.ENSP00000478570	0	0	0	0	0	0.379	0.9	0.62	0.974
HSP90AA1	HSPA1A	9606.ENSP00000335153	9606.ENSP00000364802	0	0	0	0	0.317	0.713	0.9	0.876	0.997
HSP90AA1	HSPA8	9606.ENSP00000335153	9606.ENSP00000432083	0	0	0	0	0.771	0.73	0.9	0.929	0.999
HSPA1A	MAPK1	9606.ENSP00000364802	9606.ENSP00000215832	0	0	0	0	0	0.157	0.9	0.303	0.936
HSPA1A	IFNG	9606.ENSP00000364802	9606.ENSP00000229135	0	0	0	0	0	0	0.9	0.286	0.925
HSPA1A	NR3C1	9606.ENSP00000364802	9606.ENSP00000231509	0	0	0	0	0	0.423	0.9	0.425	0.963
HSPA1A	HSPB1	9606.ENSP00000364802	9606.ENSP00000248553	0	0	0	0	0.092	0.127	0.9	0.771	0.979
HSPA1A	PABPC1	9606.ENSP00000364802	9606.ENSP00000313007	0	0	0	0	0.112	0.13	0.9	0.165	0.926
HSPA1A	HSPA5	9606.ENSP00000364802	9606.ENSP00000324173	0	0	0.448	0.965	0.226	0.805	0.9	0.761	0.984
HSPA1A	NR3C2	9606.ENSP00000364802	9606.ENSP00000350815	0	0	0	0	0	0.109	0.9	0.253	0.927

HSPA1A	PPARA	9606.ENSP00000364802	9606.ENSP00000385523	0	0	0	0	0	0.109	0.9	0.092	0.912
HSPA1A	HSPA8	9606.ENSP00000364802	9606.ENSP00000432083	0	0	0.449	0.983	0.061	0.387	0.9	0.812	0.938
HSPA5	HSPA8	9606.ENSP00000324173	9606.ENSP00000432083	0	0	0.448	0.966	0.241	0.378	0.9	0.798	0.950
HSPA8	NR3C1	9606.ENSP00000432083	9606.ENSP00000231509	0	0	0	0	0	0.166	0.9	0.386	0.944
HSPA8	HSPB1	9606.ENSP00000432083	9606.ENSP00000248553	0	0	0	0	0.065	0.42	0.9	0.742	0.984
HSPA8	SORT1	9606.ENSP00000432083	9606.ENSP00000256637	0	0	0	0	0.053	0	0.9	0.104	0.907
HSPA8	SF3B3	9606.ENSP00000432083	9606.ENSP00000305790	0	0	0	0	0.105	0.104	0.9	0.07	0.915
HSPA8	PABPC1	9606.ENSP00000432083	9606.ENSP00000313007	0	0	0	0	0.35	0.422	0.9	0.566	0.981
HSPA8	IGF2R	9606.ENSP00000432083	9606.ENSP00000349437	0	0	0	0	0	0	0.9	0.212	0.917
HSPA8	NR3C2	9606.ENSP00000432083	9606.ENSP00000350815	0	0	0	0	0	0.109	0.9	0.163	0.919
HSPB1	PABPC1	9606.ENSP00000248553	9606.ENSP00000313007	0	0	0	0	0	0	0.9	0.238	0.92
HTR1B	OPRM1	9606.ENSP00000358963	9606.ENSP00000394624	0	0	0	0.65	0.061	0	0.9	0.533	0.919
HTR2A	SLC6A4	9606.ENSP00000437737	9606.ENSP00000261707	0	0	0	0	0	0.073	0	0.904	0.907
HTR2A	PIK3CA	9606.ENSP00000437737	9606.ENSP00000263967	0	0	0	0	0	0.05	0.9	0	0.900
HTR2A	PIK3R1	9606.ENSP00000437737	9606.ENSP00000428056	0	0	0	0	0.065	0.057	0.9	0	0.904
HTR7	SLC6A4	9606.ENSP00000337949	9606.ENSP00000261707	0	0	0	0	0.049	0.073	0	0.91	0.913
ICAM1	IL1B	9606.ENSP00000264832	9606.ENSP00000263341	0	0	0	0	0.154	0	0	0.916	0.926
ICAM1	STAT3	9606.ENSP00000264832	9606.ENSP00000264657	0	0	0	0	0.084	0	0.9	0.749	0.975
ICAM1	VCAM1	9606.ENSP00000264832	9606.ENSP00000294728	0	0	0	0	0.069	0	0	0.938	0.940
ICAM1	IL6	9606.ENSP00000264832	9606.ENSP00000385675	0	0	0	0	0.152	0	0	0.947	0.953
ICAM1	TNF	9606.ENSP00000264832	9606.ENSP00000398698	0	0	0	0	0.119	0	0	0.957	0.960
ICAM1	IL10	9606.ENSP00000264832	9606.ENSP00000412237	0	0	0	0	0	0	0.9	0.858	0.985
IFNG	IL2	9606.ENSP00000229135	9606.ENSP00000226730	0	0	0	0	0.087	0	0	0.935	0.938
IFNG	IL1B	9606.ENSP00000229135	9606.ENSP00000263341	0	0	0	0	0.082	0	0	0.898	0.902
IFNG	PTPN1	9606.ENSP00000229135	9606.ENSP00000360683	0	0	0	0	0	0	0.9	0.112	0.907
IFNG	PRKCD	9606.ENSP00000229135	9606.ENSP00000378217	0	0	0	0	0	0	0.9	0.187	0.915

IFNG	PIK3CA	9606.ENSP00000229135	9606.ENSP00000263967	0	0	0	0	0	0	0.9	0.185	0.915
IFNG	TERT	9606.ENSP00000229135	9606.ENSP00000309572	0	0	0	0	0	0	0.9	0.215	0.918
IFNG	PIK3R1	9606.ENSP00000229135	9606.ENSP00000428056	0	0	0	0	0	0	0.9	0.217	0.918
IFNG	IL6	9606.ENSP00000229135	9606.ENSP00000385675	0	0	0	0	0.064	0	0	0.937	0.939
IFNG	IL10	9606.ENSP00000229135	9606.ENSP00000412237	0	0	0	0	0.064	0	0	0.944	0.946
IFNG	JUN	9606.ENSP00000229135	9606.ENSP00000360266	0	0	0	0	0	0	0.9	0.518	0.949
IFNG	RELA	9606.ENSP00000229135	9606.ENSP00000384273	0	0	0	0	0	0	0.9	0.614	0.959
IFNG	TNF	9606.ENSP00000229135	9606.ENSP00000398698	0	0	0	0	0.152	0.379	0	0.957	0.975
IFNG	STAT1	9606.ENSP00000229135	9606.ENSP00000354394	0	0	0	0	0.063	0	0.9	0.898	0.989
IGF1R	MAPK1	9606.ENSP00000268035	9606.ENSP00000215832	0	0	0	0.57	0	0.146	0.9	0.638	0.934
IGF1R	PIK3CA	9606.ENSP00000268035	9606.ENSP00000263967	0	0	0	0	0.062	0.124	0.9	0.837	0.984
IGF1R	VEGFA	9606.ENSP00000268035	9606.ENSP00000478570	0	0	0	0	0	0	0.6	0.796	0.915
IGF1R	PRKCD	9606.ENSP00000268035	9606.ENSP00000378217	0	0	0	0.551	0.062	0.334	0.9	0.488	0.946
IGF1R	PIK3R1	9606.ENSP00000268035	9606.ENSP00000428056	0	0	0	0	0.049	0.586	0.9	0.546	0.979
IGF1R	INSR	9606.ENSP00000268035	9606.ENSP00000303830	0	0	0	0.957	0.062	0.974	0.8	0.869	0.994
IGF1R	IGF2	9606.ENSP00000268035	9606.ENSP00000391826	0	0	0	0	0	0.522	0.9	0.951	0.997
IGF1R	PTPN1	9606.ENSP00000268035	9606.ENSP00000360683	0	0	0	0	0.062	0.973	0.9	0.656	0.999
IGF2	MMP2	9606.ENSP00000391826	9606.ENSP00000219070	0	0	0	0	0.165	0	0.9	0.399	0.945
IGF2	SERPINE1	9606.ENSP00000391826	9606.ENSP00000223095	0	0	0	0	0.088	0	0.9	0.311	0.931
IGF2	INSR	9606.ENSP00000391826	9606.ENSP00000303830	0	0	0	0	0	0.522	0.6	0.776	0.953
IGF2	PLG	9606.ENSP00000391826	9606.ENSP00000308938	0	0	0	0	0.061	0.379	0.9	0.361	0.957
IGF2	MMP1	9606.ENSP00000391826	9606.ENSP00000322788	0	0	0	0	0.076	0	0.9	0.257	0.925
IGF2	IGF2R	9606.ENSP00000391826	9606.ENSP00000349437	0	0	0	0	0	0.825	0	0.966	0.993
IGF2	IGFBP3	9606.ENSP00000391826	9606.ENSP00000370473	0	0	0	0	0.155	0.379	0.9	0.938	0.996
IGF2	VEGFA	9606.ENSP00000391826	9606.ENSP00000478570	0	0	0	0	0.069	0	0.9	0.809	0.980
IGF2R	SORT1	9606.ENSP00000349437	9606.ENSP00000256637	0	0	0	0	0.061	0.108	0.9	0.667	0.968

IGFBP3	MMP2	9606.ENSP00000370473	9606.ENSP00000219070	0	0	0	0	0.196	0.379	0.9	0.413	0.966
IGFBP3	PLG	9606.ENSP00000370473	9606.ENSP00000308938	0	0	0	0	0	0.379	0.9	0.64	0.975
IGFBP3	MMP1	9606.ENSP00000370473	9606.ENSP00000322788	0	0	0	0	0.142	0.379	0.9	0.327	0.959
IGFBP3	MAPK8	9606.ENSP00000370473	9606.ENSP00000378974	0	0	0	0	0	0	0.9	0.292	0.926
IGFBP3	SPP1	9606.ENSP00000370473	9606.ENSP00000378517	0	0	0	0	0.07	0	0.9	0.359	0.935
IGFBP3	IL6	9606.ENSP00000370473	9606.ENSP00000385675	0	0	0	0	0.076	0	0.9	0.536	0.953
IKBKB	TP63	9606.ENSP00000430684	9606.ENSP00000264731	0	0	0	0	0	0.379	0.9	0.139	0.941
IKBKB	PRKCB	9606.ENSP00000430684	9606.ENSP00000305355	0	0	0	0.565	0	0.697	0.9	0.297	0.972
IKBKB	PRKCE	9606.ENSP00000430684	9606.ENSP00000306124	0	0	0	0.559	0.062	0.398	0.9	0.304	0.946
IKBKB	MTOR	9606.ENSP00000430684	9606.ENSP00000354558	0	0	0	0	0.058	0.882	0	0.316	0.917
IKBKB	RELA	9606.ENSP00000430684	9606.ENSP00000384273	0	0	0	0	0.063	0.871	0.9	0.831	0.997
IKBKB	TNF	9606.ENSP00000430684	9606.ENSP00000398698	0	0	0	0	0.061	0.993	0.9	0.684	0.999
IKBKB	PRKCA	9606.ENSP00000430684	9606.ENSP00000408695	0	0	0	0.567	0	0.358	0.9	0.268	0.939
IL10	IL2	9606.ENSP00000412237	9606.ENSP00000226730	0	0	0	0	0	0	0	0.956	0.957
IL10	IL1A	9606.ENSP00000412237	9606.ENSP00000263339	0	0	0	0	0.063	0	0.9	0.738	0.973
IL10	IL1B	9606.ENSP00000412237	9606.ENSP00000263341	0	0	0	0	0.085	0	0.9	0.96	0.996
IL10	STAT3	9606.ENSP00000412237	9606.ENSP00000264657	0	0	0	0	0	0.429	0.9	0.933	0.995
IL10	TLR9	9606.ENSP00000412237	9606.ENSP00000353874	0	0	0	0	0.055	0.157	0	0.911	0.923
IL10	IL6	9606.ENSP00000412237	9606.ENSP00000385675	0	0	0	0	0.077	0	0.9	0.974	0.997
IL10	TNF	9606.ENSP00000412237	9606.ENSP00000398698	0	0	0	0	0.097	0	0.9	0.974	0.997
IL1A	STAT3	9606.ENSP00000263339	9606.ENSP00000264657	0	0	0	0	0	0	0.9	0.354	0.932
IL1A	RELA	9606.ENSP00000263339	9606.ENSP00000384273	0	0	0	0	0	0	0.9	0.355	0.932
IL1A	JUN	9606.ENSP00000263339	9606.ENSP00000360266	0	0	0	0	0	0	0.9	0.442	0.941
IL1A	TNF	9606.ENSP00000263339	9606.ENSP00000398698	0	0	0	0	0.296	0	0	0.923	0.944
IL1A	IL6	9606.ENSP00000263339	9606.ENSP00000385675	0	0	0	0	0.375	0	0	0.918	0.946
IL1A	IL1B	9606.ENSP00000263339	9606.ENSP00000263341	0	0	0	0	0.753	0	0.9	0.921	0.997

IL1B	IL2	9606.ENSP00000263341	9606.ENSP00000226730	0	0	0	0	0	0	0	0.9	0.900
IL1B	PIK3R1	9606.ENSP00000263341	9606.ENSP00000428056	0	0	0	0	0	0	0.9	0.138	0.910
IL1B	MMP9	9606.ENSP00000263341	9606.ENSP00000361405	0	0	0	0	0.2	0	0	0.895	0.913
IL1B	PIK3CA	9606.ENSP00000263341	9606.ENSP00000263967	0	0	0	0	0	0	0.9	0.185	0.915
IL1B	RELA	9606.ENSP00000263341	9606.ENSP00000384273	0	0	0	0	0	0	0.9	0.559	0.954
IL1B	JUN	9606.ENSP00000263341	9606.ENSP00000360266	0	0	0	0	0.052	0	0.8	0.82	0.963
IL1B	STAT3	9606.ENSP00000263341	9606.ENSP00000264657	0	0	0	0	0	0	0.9	0.669	0.965
IL1B	PTGS2	9606.ENSP00000263341	9606.ENSP00000356438	0	0	0	0	0.561	0	0	0.943	0.974
IL1B	IL6	9606.ENSP00000263341	9606.ENSP00000385675	0	0	0	0	0.43	0	0	0.973	0.984
IL1B	TNF	9606.ENSP00000263341	9606.ENSP00000398698	0	0	0	0	0.441	0	0	0.981	0.989
IL2	MAPK1	9606.ENSP00000226730	9606.ENSP00000215832	0	0	0	0	0	0	0.9	0.559	0.954
IL2	PRKCE	9606.ENSP00000226730	9606.ENSP00000306124	0	0	0	0	0	0	0.9	0.125	0.908
IL2	PRKCB	9606.ENSP00000226730	9606.ENSP00000305355	0	0	0	0	0	0	0.9	0.227	0.919
IL2	PIK3CA	9606.ENSP00000226730	9606.ENSP00000263967	0	0	0	0	0	0	0.9	0.228	0.919
IL2	PIK3R1	9606.ENSP00000226730	9606.ENSP00000428056	0	0	0	0	0	0	0.9	0.232	0.919
IL2	TNF	9606.ENSP00000226730	9606.ENSP00000398698	0	0	0	0	0	0	0	0.956	0.957
IL2	TERT	9606.ENSP00000226730	9606.ENSP00000309572	0	0	0	0	0	0	0.9	0.599	0.958
IL2	MTOR	9606.ENSP00000226730	9606.ENSP00000354558	0	0	0	0	0	0	0.9	0.615	0.959
IL2	IL6	9606.ENSP00000226730	9606.ENSP00000385675	0	0	0	0	0	0	0	0.958	0.959
IL2	STAT1	9606.ENSP00000226730	9606.ENSP00000354394	0	0	0	0	0	0	0.9	0.672	0.965
IL2	STAT3	9606.ENSP00000226730	9606.ENSP00000264657	0	0	0	0	0	0	0.9	0.705	0.969
IL2	JUN	9606.ENSP00000226730	9606.ENSP00000360266	0	0	0	0	0	0.313	0.9	0.621	0.971
IL2	MAPK8	9606.ENSP00000226730	9606.ENSP00000378974	0	0	0	0	0	0	0.9	0.769	0.975
IL2	RELA	9606.ENSP00000226730	9606.ENSP00000384273	0	0	0	0	0	0.342	0.9	0.694	0.978
IL6	MAPK1	9606.ENSP00000385675	9606.ENSP00000215832	0	0	0	0	0	0	0.9	0.834	0.982
IL6	STAT3	9606.ENSP00000385675	9606.ENSP00000264657	0	0	0	0	0	0.269	0.9	0.97	0.997

IL6	TLR9	9606.ENSP00000385675	9606.ENSP00000353874	0	0	0	0	0	0	0	0.905	0.905
IL6	STAT1	9606.ENSP00000385675	9606.ENSP00000354394	0	0	0	0	0.058	0.163	0.9	0.818	0.983
IL6	PTGS2	9606.ENSP00000385675	9606.ENSP00000356438	0	0	0	0	0.314	0	0	0.922	0.945
IL6	JUN	9606.ENSP00000385675	9606.ENSP00000360266	0	0	0	0	0.053	0	0.9	0.85	0.984
IL6	MMP9	9606.ENSP00000385675	9606.ENSP00000361405	0	0	0	0	0.074	0	0	0.92	0.923
IL6	SPP1	9606.ENSP00000385675	9606.ENSP00000378517	0	0	0	0	0.06	0	0.9	0.793	0.978
IL6	RELA	9606.ENSP00000385675	9606.ENSP00000384273	0	0	0	0	0	0.223	0.9	0.804	0.983
IL6	VEGFA	9606.ENSP00000385675	9606.ENSP00000478570	0	0	0	0	0.063	0	0	0.957	0.959
IL6	TNF	9606.ENSP00000385675	9606.ENSP00000398698	0	0	0	0	0.123	0	0	0.982	0.984
INSR	PIK3CA	9606.ENSP00000303830	9606.ENSP00000263967	0	0	0	0	0.062	0.124	0.9	0.468	0.950
INSR	PTPRA	9606.ENSP00000303830	9606.ENSP00000369756	0	0	0	0	0.062	0.16	0.9	0.084	0.918
INSR	PIK3R1	9606.ENSP00000303830	9606.ENSP00000428056	0	0	0	0	0.061	0.472	0.9	0.492	0.971
INSR	PTPN1	9606.ENSP00000303830	9606.ENSP00000360683	0	0	0	0	0.062	0.972	0.9	0.418	0.998
ITGB1	PIK3CA	9606.ENSP00000379350	9606.ENSP00000263967	0	0	0	0	0.064	0	0.9	0.208	0.919
ITGB1	STAT3	9606.ENSP00000379350	9606.ENSP00000264657	0	0	0	0	0.063	0	0.9	0.35	0.933
ITGB1	VCAM1	9606.ENSP00000379350	9606.ENSP00000294728	0	0	0	0	0.129	0.379	0.9	0.73	0.983
ITGB1	MET	9606.ENSP00000379350	9606.ENSP00000317272	0	0	0	0	0.092	0.157	0.9	0.358	0.944
ITGB1	STAT1	9606.ENSP00000379350	9606.ENSP00000354394	0	0	0	0	0.061	0	0.9	0.205	0.918
ITGB1	SPP1	9606.ENSP00000379350	9606.ENSP00000378517	0	0	0	0	0.061	0.379	0.9	0.709	0.980
ITGB1	PIK3R1	9606.ENSP00000379350	9606.ENSP00000428056	0	0	0	0	0	0	0.9	0.187	0.915
ITGB1	VEGFA	9606.ENSP00000379350	9606.ENSP00000478570	0	0	0	0	0	0.064	0.9	0.575	0.956
JUN	MAPK1	9606.ENSP00000360266	9606.ENSP00000215832	0	0	0	0	0	0.411	0.9	0.952	0.996
JUN	NR3C1	9606.ENSP00000360266	9606.ENSP00000231509	0	0	0	0	0	0.472	0.9	0.739	0.985
JUN	STAT3	9606.ENSP00000360266	9606.ENSP00000264657	0	0	0	0	0.055	0.476	0	0.852	0.921
JUN	PPARG	9606.ENSP00000360266	9606.ENSP00000287820	0	0	0	0	0	0.144	0.9	0.533	0.956
JUN	NOS3	9606.ENSP00000360266	9606.ENSP00000297494	0	0	0	0	0	0	0.9	0.542	0.952

JUN	NOS2	9606.ENSP00000360266	9606.ENSP00000327251	0	0	0	0	0	0	0.9	0.584	0.956
JUN	PPARA	9606.ENSP00000360266	9606.ENSP00000385523	0	0	0	0	0	0.082	0.9	0.385	0.938
JUN	NFE2L2	9606.ENSP00000360266	9606.ENSP00000380252	0	0	0	0	0	0.4	0.9	0.578	0.972
JUN	RELA	9606.ENSP00000360266	9606.ENSP00000384273	0	0	0	0	0.069	0.457	0.9	0.667	0.980
JUN	VEGFA	9606.ENSP00000360266	9606.ENSP00000478570	0	0	0	0	0.066	0.261	0.9	0.794	0.983
JUN	TNF	9606.ENSP00000360266	9606.ENSP00000398698	0	0	0	0	0	0	0.9	0.871	0.986
JUN	MYC	9606.ENSP00000360266	9606.ENSP00000479618	0	0	0	0	0.063	0.379	0.9	0.878	0.992
JUN	MAPK8	9606.ENSP00000360266	9606.ENSP00000378974	0	0	0	0	0	0.788	0.9	0.985	0.999
MAOA	MAOB	9606.ENSP00000340684	9606.ENSP00000367309	0	0	0.448	0.976	0.18	0.685	0.8	0.931	0.945
MAPK1	RXRG	9606.ENSP00000215832	9606.ENSP00000352900	0	0	0	0	0	0.073	0.9	0.051	0.904
MAPK1	TTR	9606.ENSP00000215832	9606.ENSP00000237014	0	0	0	0	0	0	0.9	0.185	0.915
MAPK1	PIN1	9606.ENSP00000215832	9606.ENSP00000247970	0	0	0	0	0.055	0	0.9	0.193	0.917
MAPK1	PRKCB	9606.ENSP00000215832	9606.ENSP00000305355	0	0	0	0.59	0.056	0.158	0.9	0.414	0.927
MAPK1	MAPT	9606.ENSP00000215832	9606.ENSP00000340820	0	0	0	0	0	0	0.8	0.662	0.929
MAPK1	MET	9606.ENSP00000215832	9606.ENSP00000317272	0	0	0	0.589	0.059	0.146	0.9	0.481	0.929
MAPK1	RARA	9606.ENSP00000215832	9606.ENSP00000254066	0	0	0	0	0	0.317	0.9	0.188	0.939
MAPK1	RXRA	9606.ENSP00000215832	9606.ENSP00000419692	0	0	0	0	0	0.363	0.9	0.154	0.941
MAPK1	PRKCA	9606.ENSP00000215832	9606.ENSP00000408695	0	0	0	0.594	0.056	0.261	0.9	0.575	0.941
MAPK1	MPO	9606.ENSP00000215832	9606.ENSP00000225275	0	0	0	0	0	0.077	0.9	0.445	0.944
MAPK1	MAPK8	9606.ENSP00000215832	9606.ENSP00000378974	0	0	0.386	0.886	0.081	0.384	0.9	0.98	0.947
MAPK1	PPARA	9606.ENSP00000215832	9606.ENSP00000385523	0	0	0	0	0	0.399	0.9	0.264	0.951
MAPK1	NR4A1	9606.ENSP00000215832	9606.ENSP00000440864	0	0	0	0	0	0.423	0.8	0.61	0.951
MAPK1	RELA	9606.ENSP00000215832	9606.ENSP00000384273	0	0	0	0	0	0	0.9	0.541	0.952
MAPK1	TERT	9606.ENSP00000215832	9606.ENSP00000309572	0	0	0	0	0.056	0	0.9	0.569	0.955
MAPK1	PRKCE	9606.ENSP00000215832	9606.ENSP00000306124	0	0	0	0.595	0.062	0.522	0.9	0.421	0.959
MAPK1	NR3C1	9606.ENSP00000215832	9606.ENSP00000231509	0	0	0	0	0	0.399	0.9	0.413	0.961

MAPK1	PRKCD	9606.ENSP00000215832	9606.ENSP00000378217	0	0	0	0.589	0.062	0.522	0.9	0.534	0.961
MAPK1	RUNX2	9606.ENSP00000215832	9606.ENSP00000360493	0	0	0	0	0	0	0.9	0.74	0.972
MAPK1	STAT1	9606.ENSP00000215832	9606.ENSP00000354394	0	0	0	0	0.06	0.263	0.9	0.649	0.972
MAPK1	MYC	9606.ENSP00000215832	9606.ENSP00000479618	0	0	0	0	0	0.379	0.9	0.798	0.986
MAPK1	STAT3	9606.ENSP00000215832	9606.ENSP00000264657	0	0	0	0	0.06	0.384	0.9	0.837	0.989
MAPK8	NR3C1	9606.ENSP00000378974	9606.ENSP00000231509	0	0	0	0	0.054	0.379	0.9	0.476	0.965
MAPK8	RARA	9606.ENSP00000378974	9606.ENSP00000254066	0	0	0	0	0	0	0.9	0.218	0.918
MAPK8	SORT1	9606.ENSP00000378974	9606.ENSP00000256637	0	0	0	0	0.079	0	0.9	0.267	0.926
MAPK8	STAT3	9606.ENSP00000378974	9606.ENSP00000264657	0	0	0	0	0	0.379	0	0.854	0.906
MAPK8	PRKCB	9606.ENSP00000378974	9606.ENSP00000305355	0	0	0	0.576	0.049	0	0.9	0.478	0.92
MAPK8	MET	9606.ENSP00000378974	9606.ENSP00000317272	0	0	0	0.56	0.062	0.073	0.9	0.447	0.923
MAPK8	MAPT	9606.ENSP00000378974	9606.ENSP00000340820	0	0	0	0	0.064	0.294	0.9	0.479	0.961
MAPK8	TNF	9606.ENSP00000378974	9606.ENSP00000398698	0	0	0	0	0	0	0	0.91	0.910
MAPK8	RXRA	9606.ENSP00000378974	9606.ENSP00000419692	0	0	0	0	0	0	0.9	0.262	0.923
MAPK8	RELA	9606.ENSP00000378974	9606.ENSP00000384273	0	0	0	0	0	0	0.8	0.769	0.952
MET	PIK3CA	9606.ENSP00000317272	9606.ENSP00000263967	0	0	0	0	0.063	0.124	0.9	0.586	0.961
MET	STAT3	9606.ENSP00000317272	9606.ENSP00000264657	0	0	0	0	0.062	0.432	0.9	0.647	0.978
MET	VEGFA	9606.ENSP00000317272	9606.ENSP00000478570	0	0	0	0	0.067	0	0.6	0.826	0.929
MET	PIK3R1	9606.ENSP00000317272	9606.ENSP00000428056	0	0	0	0	0	0.407	0.9	0.262	0.952
MET	PTPN1	9606.ENSP00000317272	9606.ENSP00000360683	0	0	0	0	0.062	0.538	0.9	0.323	0.966
MMP1	MMP2	9606.ENSP00000322788	9606.ENSP00000219070	0	0	0	0.869	0.154	0	0.9	0.921	0.922
MMP1	MMP13	9606.ENSP00000322788	9606.ENSP00000260302	0	0	0	0.935	0.077	0	0.9	0.863	0.909
MMP1	STAT3	9606.ENSP00000322788	9606.ENSP00000264657	0	0	0	0	0	0	0.9	0.674	0.966
MMP1	PLG	9606.ENSP00000322788	9606.ENSP00000308938	0	0	0	0	0.063	0	0.9	0.615	0.960
MMP1	MMP9	9606.ENSP00000322788	9606.ENSP00000361405	0	0	0	0.75	0.518	0	0.9	0.91	0.961
MMP13	MMP2	9606.ENSP00000260302	9606.ENSP00000219070	0	0	0	0.89	0.061	0	0.9	0.835	0.911

MMP13	MMP9	9606.ENSP00000260302	9606.ENSP00000361405	0	0	0	0.797	0.064	0	0.9	0.878	0.919
MMP13	PLG	9606.ENSP00000260302	9606.ENSP00000308938	0	0	0	0	0.063	0	0.9	0.507	0.949
MMP2	MMP9	9606.ENSP00000219070	9606.ENSP00000361405	0	0	0	0.929	0.061	0	0.9	0.958	0.908
MMP2	STAT3	9606.ENSP00000219070	9606.ENSP00000264657	0	0	0	0	0	0	0.9	0.799	0.979
MMP2	VEGFA	9606.ENSP00000219070	9606.ENSP00000478570	0	0	0	0	0.065	0	0.8	0.899	0.979
MMP9	STAT3	9606.ENSP00000361405	9606.ENSP00000264657	0	0	0	0	0	0	0.9	0.824	0.981
MMP9	PLG	9606.ENSP00000361405	9606.ENSP00000308938	0	0	0	0	0.063	0.379	0.9	0.863	0.991
MMP9	TNF	9606.ENSP00000361405	9606.ENSP00000398698	0	0	0	0	0.176	0	0	0.935	0.944
MMP9	VEGFA	9606.ENSP00000361405	9606.ENSP00000478570	0	0	0	0	0	0	0.8	0.946	0.989
MPI	PFKFB3	9606.ENSP00000318318	9606.ENSP00000443319	0	0	0	0	0.063	0.05	0.9	0.173	0.916
MPO	PRKCD	9606.ENSP00000225275	9606.ENSP00000378217	0	0	0	0	0	0.064	0.9	0.069	0.905
MPO	TTR	9606.ENSP00000225275	9606.ENSP00000237014	0	0	0	0	0	0	0.9	0.242	0.921
MPO	PTGS1	9606.ENSP00000225275	9606.ENSP00000354612	0	0	0	0	0.076	0	0.9	0.408	0.940
MTOR	PIK3CA	9606.ENSP00000354558	9606.ENSP00000263967	0	0	0	0.541	0.061	0.186	0.9	0.73	0.944
MTOR	STAT3	9606.ENSP00000354558	9606.ENSP00000264657	0	0	0	0	0.049	0.379	0.9	0.835	0.989
MTOR	PPARG	9606.ENSP00000354558	9606.ENSP00000287820	0	0	0	0	0	0	0.8	0.529	0.901
MTOR	TERT	9606.ENSP00000354558	9606.ENSP00000309572	0	0	0	0	0	0.464	0.9	0.371	0.963
MTOR	RELA	9606.ENSP00000354558	9606.ENSP00000384273	0	0	0	0	0	0	0.9	0.33	0.930
MTOR	PRKCA	9606.ENSP00000354558	9606.ENSP00000408695	0	0	0	0	0	0.234	0.9	0.372	0.947
MTOR	PIK3R1	9606.ENSP00000354558	9606.ENSP00000428056	0	0	0	0	0.061	0.269	0.9	0.491	0.960
MYC	PIN1	9606.ENSP00000479618	9606.ENSP00000247970	0	0	0	0	0	0.379	0.9	0.268	0.950
MYC	PIK3CA	9606.ENSP00000479618	9606.ENSP00000263967	0	0	0	0	0	0.201	0.9	0.648	0.969
MYC	STAT3	9606.ENSP00000479618	9606.ENSP00000264657	0	0	0	0	0	0.379	0.9	0.859	0.990
MYC	TERT	9606.ENSP00000479618	9606.ENSP00000309572	0	0	0	0	0.077	0.139	0.9	0.884	0.989
MYC	NOS2	9606.ENSP00000479618	9606.ENSP00000327251	0	0	0	0	0	0	0.9	0.222	0.918
MYC	RELA	9606.ENSP00000479618	9606.ENSP00000384273	0	0	0	0	0	0.379	0.9	0.587	0.972

MYC	PPARA	9606.ENSP00000479618	9606.ENSP00000385523	0	0	0	0	0	0	0.9	0.355	0.932
MYC	TNF	9606.ENSP00000479618	9606.ENSP00000398698	0	0	0	0	0.062	0	0.9	0.769	0.976
MYC	RXRA	9606.ENSP00000479618	9606.ENSP00000419692	0	0	0	0	0	0	0.9	0.299	0.926
MYC	PIK3R1	9606.ENSP00000479618	9606.ENSP00000428056	0	0	0	0	0	0.133	0.9	0.421	0.945
NCF1	NOX4	9606.ENSP00000289473	9606.ENSP00000263317	0	0	0	0	0.055	0	0.36	0.898	0.933
NCF1	PRKCA	9606.ENSP00000289473	9606.ENSP00000408695	0	0	0	0	0	0.336	0.8	0.31	0.900
NCF1	PRKCB	9606.ENSP00000289473	9606.ENSP00000305355	0	0	0	0	0.088	0.379	0.8	0.297	0.909
NCF1	VCAM1	9606.ENSP00000289473	9606.ENSP00000294728	0	0	0	0	0.062	0	0.9	0.444	0.943
NFE2L2	PRKCB	9606.ENSP00000380252	9606.ENSP00000305355	0	0	0	0	0	0	0.9	0.111	0.907
NFE2L2	NQO1	9606.ENSP00000380252	9606.ENSP00000319788	0	0	0	0	0	0	0	0.94	0.941
NFE2L2	PRKCA	9606.ENSP00000380252	9606.ENSP00000408695	0	0	0	0	0	0.379	0.9	0.145	0.942
NOS2	STAT3	9606.ENSP00000327251	9606.ENSP00000264657	0	0	0	0	0	0	0.9	0.575	0.955
NOS2	PTGS2	9606.ENSP00000327251	9606.ENSP00000356438	0	0	0	0	0.063	0.299	0	0.868	0.906
NOS2	RXRA	9606.ENSP00000327251	9606.ENSP00000419692	0	0	0	0	0	0.091	0.9	0.087	0.909
NOS2	PPARA	9606.ENSP00000327251	9606.ENSP00000385523	0	0	0	0	0	0.091	0.9	0.211	0.922
NOS2	RELA	9606.ENSP00000327251	9606.ENSP00000384273	0	0	0	0	0	0.343	0.9	0.327	0.951
NOS2	TNF	9606.ENSP00000327251	9606.ENSP00000398698	0	0	0	0	0.063	0	0.9	0.844	0.984
NOS3	PRKCD	9606.ENSP00000297494	9606.ENSP00000378217	0	0	0	0	0	0.08	0.9	0.528	0.952
NOS3	VEGFA	9606.ENSP00000297494	9606.ENSP00000478570	0	0	0	0	0.062	0	0.9	0.934	0.993
NOX4	PTPN1	9606.ENSP00000263317	9606.ENSP00000360683	0	0	0	0	0.05	0	0.9	0.515	0.949
NQO1	TP63	9606.ENSP00000319788	9606.ENSP00000264731	0	0	0	0	0	0	0.9	0.066	0.902
NR1I2	RXRA	9606.ENSP00000336528	9606.ENSP00000419692	0	0	0	0.631	0.061	0.89	0	0.639	0.917
NR3C1	PIK3CA	9606.ENSP00000231509	9606.ENSP00000263967	0	0	0	0	0.081	0	0.9	0.19	0.919
NR3C1	PIK3R1	9606.ENSP00000231509	9606.ENSP00000428056	0	0	0	0	0.062	0.081	0.9	0.211	0.922
NR3C1	RXRA	9606.ENSP00000231509	9606.ENSP00000419692	0	0	0	0.632	0	0	0.9	0.641	0.923
NR3C1	STAT1	9606.ENSP00000231509	9606.ENSP00000354394	0	0	0	0	0.065	0.053	0.9	0.4	0.939

NR3C1	NR3C2	9606.ENSP00000231509	9606.ENSP00000350815	0	0	0	0.861	0	0.379	0.9	0.942	0.943
NR3C1	RARA	9606.ENSP00000231509	9606.ENSP00000254066	0	0	0	0.614	0	0.43	0.9	0.377	0.948
NR3C1	TNF	9606.ENSP00000231509	9606.ENSP00000398698	0	0	0	0	0	0	0.9	0.577	0.955
NR3C1	RELA	9606.ENSP00000231509	9606.ENSP00000384273	0	0	0	0	0	0.393	0.9	0.443	0.963
NR4A1	RXRG	9606.ENSP00000440864	9606.ENSP00000352900	0	0	0	0.749	0	0.42	0.9	0.223	0.942
NR4A1	RXRA	9606.ENSP00000440864	9606.ENSP00000419692	0	0	0	0.752	0	0.47	0.9	0.411	0.950
OPRM1	STAT3	9606.ENSP00000394624	9606.ENSP00000264657	0	0	0	0	0	0	0.9	0.222	0.918
PIK3CA	RELA	9606.ENSP00000263967	9606.ENSP00000384273	0	0	0	0	0	0	0.9	0.26	0.922
PIK3CA	PTPN1	9606.ENSP00000263967	9606.ENSP00000360683	0	0	0	0	0.061	0.172	0.9	0.137	0.923
PIK3CA	PIK3CG	9606.ENSP00000263967	9606.ENSP00000352121	0	0	0	0.815	0.061	0.154	0.9	0.729	0.925
PIK3CA	STAT3	9606.ENSP00000263967	9606.ENSP00000264657	0	0	0	0	0.049	0	0.9	0.454	0.943
PIK3CA	VEGFA	9606.ENSP00000263967	9606.ENSP00000478570	0	0	0	0	0	0	0.9	0.535	0.951
PIK3CA	PRKCD	9606.ENSP00000263967	9606.ENSP00000378217	0	0	0	0	0.064	0.459	0.9	0.198	0.954
PIK3CA	PIK3R1	9606.ENSP00000263967	9606.ENSP00000428056	0	0	0	0	0.082	0.974	0.9	0.899	0.999
PIK3CG	PIK3R1	9606.ENSP00000352121	9606.ENSP00000428056	0	0	0	0	0.061	0.446	0.9	0.691	0.981
PIK3R1	STAT3	9606.ENSP00000428056	9606.ENSP00000264657	0	0	0	0	0	0.513	0.9	0.599	0.978
PIK3R1	PTPN1	9606.ENSP00000428056	9606.ENSP00000360683	0	0	0	0	0	0.123	0.9	0.234	0.927
PIK3R1	PRKCD	9606.ENSP00000428056	9606.ENSP00000378217	0	0	0	0	0	0.079	0.9	0.265	0.926
PIK3R1	RELA	9606.ENSP00000428056	9606.ENSP00000384273	0	0	0	0	0	0	0.9	0.236	0.920
PIK3R1	VEGFA	9606.ENSP00000428056	9606.ENSP00000478570	0	0	0	0	0	0	0.9	0.313	0.928
PLG	SERPINE1	9606.ENSP00000308938	9606.ENSP00000223095	0	0	0	0	0.063	0.435	0.9	0.978	0.998
PLG	VEGFA	9606.ENSP00000308938	9606.ENSP00000478570	0	0	0	0	0	0	0.9	0.708	0.969
PPARA	RXRG	9606.ENSP00000385523	9606.ENSP00000352900	0	0	0	0.696	0.062	0.399	0.9	0.474	0.947
PPARA	RELA	9606.ENSP00000385523	9606.ENSP00000384273	0	0	0	0	0	0.393	0.9	0.21	0.947
PPARA	TNF	9606.ENSP00000385523	9606.ENSP00000398698	0	0	0	0	0	0	0.9	0.538	0.951
PPARA	RXRA	9606.ENSP00000385523	9606.ENSP00000419692	0	0	0	0.695	0.062	0.478	0.9	0.715	0.958

PPARD	RXRG	9606.ENSP00000310928	9606.ENSP00000352900	0	0	0	0.689	0	0.399	0.9	0.474	0.946
PPARD	RXRA	9606.ENSP00000310928	9606.ENSP00000419692	0	0	0	0.705	0.062	0.467	0.9	0.604	0.955
PPARG	RELA	9606.ENSP00000287820	9606.ENSP00000384273	0	0	0	0	0	0.472	0.9	0.348	0.962
PPARG	SLC2A4	9606.ENSP00000287820	9606.ENSP00000320935	0	0	0	0	0.061	0	0.9	0.736	0.973
PPARG	TNF	9606.ENSP00000287820	9606.ENSP00000398698	0	0	0	0	0	0	0.9	0.827	0.982
PPARG	RXRG	9606.ENSP00000287820	9606.ENSP00000352900	0	0	0	0.702	0	0.816	0.9	0.467	0.983
PPARG	RXRA	9606.ENSP00000287820	9606.ENSP00000419692	0	0	0	0.694	0.061	0.961	0.9	0.821	0.997
PRKCA	RARA	9606.ENSP00000408695	9606.ENSP00000254066	0	0	0	0	0	0.379	0.9	0.155	0.943
PRKCA	PRKCB	9606.ENSP00000408695	9606.ENSP00000305355	0	0	0	0.981	0	0.39	0.9	0.9	0.937
PRKCA	PRKCE	9606.ENSP00000408695	9606.ENSP00000306124	0	0	0	0.924	0.055	0.871	0.9	0.733	0.987
PRKCA	TERT	9606.ENSP00000408695	9606.ENSP00000309572	0	0	0	0	0	0.379	0.9	0.172	0.944
PRKCA	RXRG	9606.ENSP00000408695	9606.ENSP00000352900	0	0	0	0	0	0	0.9	0	0.900
PRKCA	PRKCD	9606.ENSP00000408695	9606.ENSP00000378217	0	0	0	0.891	0	0	0.9	0.731	0.907
PRKCA	RELA	9606.ENSP00000408695	9606.ENSP00000384273	0	0	0	0	0	0	0.9	0.214	0.918
PRKCA	RXRA	9606.ENSP00000408695	9606.ENSP00000419692	0	0	0	0	0	0	0.9	0.123	0.908
PRKCB	PRKCE	9606.ENSP00000305355	9606.ENSP00000306124	0	0	0	0.922	0.055	0	0.9	0.617	0.906
PRKCB	RELA	9606.ENSP00000305355	9606.ENSP00000384273	0	0	0	0	0	0	0.9	0.222	0.918
PRKCB	PRKCD	9606.ENSP00000305355	9606.ENSP00000378217	0	0	0	0.89	0	0.752	0.9	0.632	0.975
PRKCD	TTR	9606.ENSP00000378217	9606.ENSP00000237014	0	0	0	0	0	0	0.9	0.106	0.906
PRKCD	STAT3	9606.ENSP00000378217	9606.ENSP00000264657	0	0	0	0	0.061	0.394	0.9	0.321	0.956
PRKCD	PRKCE	9606.ENSP00000378217	9606.ENSP00000306124	0	0	0	0.919	0	0	0.9	0.779	0.906
PRKCD	STAT1	9606.ENSP00000378217	9606.ENSP00000354394	0	0	0	0	0.061	0.394	0.9	0.298	0.954
PRKCD	RELA	9606.ENSP00000378217	9606.ENSP00000384273	0	0	0	0	0.061	0	0.9	0.22	0.920
PRKDC	TBK1	9606.ENSP00000313420	9606.ENSP00000329967	0	0	0	0	0.058	0.124	0.9	0.173	0.922
PTGS2	STAT3	9606.ENSP00000356438	9606.ENSP00000264657	0	0	0	0	0	0	0.9	0.81	0.980
PTGS2	VEGFA	9606.ENSP00000356438	9606.ENSP00000478570	0	0	0	0	0.082	0	0	0.914	0.918

PTGS2	TNF	9606.ENSP00000356438	9606.ENSP00000398698	0	0	0	0	0.096	0	0	0.921	0.926
RARA	VDR	9606.ENSP00000254066	9606.ENSP00000447173	0	0	0	0.697	0	0	0.9	0.497	0.914
RARA	RXRG	9606.ENSP00000254066	9606.ENSP00000352900	0	0	0	0.791	0.059	0.838	0.9	0.562	0.985
RARA	RXRA	9606.ENSP00000254066	9606.ENSP00000419692	0	0	0	0.773	0.088	0.979	0.9	0.747	0.998
RELA	TBK1	9606.ENSP00000384273	9606.ENSP00000329967	0	0	0	0	0.125	0.313	0.8	0.517	0.934
RELA	TNF	9606.ENSP00000384273	9606.ENSP00000398698	0	0	0	0	0	0.428	0.9	0.849	0.990
RUNX2	VDR	9606.ENSP00000360493	9606.ENSP00000447173	0	0	0	0	0.062	0.727	0	0.738	0.927
RUNX2	SPP1	9606.ENSP00000360493	9606.ENSP00000378517	0	0	0	0	0.062	0	0	0.94	0.942
RXRA	SLC2A4	9606.ENSP00000419692	9606.ENSP00000320935	0	0	0	0	0.061	0	0.9	0.198	0.918
RXRA	RXRG	9606.ENSP00000419692	9606.ENSP00000352900	0	0	0	0.971	0	0.68	0.9	0.7	0.967
RXRA	TNF	9606.ENSP00000419692	9606.ENSP00000398698	0	0	0	0	0	0	0.9	0.215	0.918
RXRA	VDR	9606.ENSP00000419692	9606.ENSP00000447173	0	0	0	0.635	0	0.438	0.9	0.677	0.955
RXRG	VDR	9606.ENSP00000352900	9606.ENSP00000447173	0	0	0	0.647	0	0.36	0.9	0.34	0.940
SERPINE1	VEGFA	9606.ENSP00000223095	9606.ENSP00000478570	0	0	0	0	0.076	0	0.9	0.821	0.982
STAT1	STAT3	9606.ENSP00000354394	9606.ENSP00000264657	0	0	0	0.948	0.062	0.713	0.9	0.95	0.972
STAT1	VCAM1	9606.ENSP00000354394	9606.ENSP00000294728	0	0	0	0	0.062	0	0.9	0.472	0.946
STAT1	TNF	9606.ENSP00000354394	9606.ENSP00000398698	0	0	0	0	0.061	0	0.9	0.73	0.972
STAT3	VCAM1	9606.ENSP00000264657	9606.ENSP00000294728	0	0	0	0	0.061	0	0.9	0.506	0.949
STAT3	TNF	9606.ENSP00000264657	9606.ENSP00000398698	0	0	0	0	0	0	0.9	0.774	0.976
STAT3	VEGFA	9606.ENSP00000264657	9606.ENSP00000478570	0	0	0	0	0	0.434	0.9	0.916	0.994
SULT1E1	TERT	9606.ENSP00000226444	9606.ENSP00000309572	0	0	0	0	0	0	0	0.925	0.925
TNF	VCAM1	9606.ENSP00000398698	9606.ENSP00000294728	0	0	0	0	0	0	0.9	0.949	0.994
TNF	VEGFA	9606.ENSP00000398698	9606.ENSP00000478570	0	0	0	0	0	0	0	0.919	0.919
